# Dual‐Band Electrochromic Smart Window for Dynamic Switching Between Radiative Cooling and Solar Heating

**DOI:** 10.1002/advs.202504483

**Published:** 2025-05-20

**Authors:** Xinyu Zhao, Qixiang Chen, Fan Fan, Qiliang Wang, Yuehong Su, Minsu Liu, Fahua Zhu, Dongliang Zhao

**Affiliations:** ^1^ School of Energy and Environment Southeast University Nanjing Jiangsu 210096 China; ^2^ Department of Architecture and Built Environment University of Nottingham Nottingham NG7 2RD UK; ^3^ ARC Research Hub for Smart Process Design and Control Department of Chemical Engineering Monash University Melbourne VIC 3800 Australia; ^4^ State Key Laboratory of Low‐carbon Smart Coal‐fired Power Generation and Ultra‐clean Emission China Energy Science and Technology Research Institute Co., Ltd. Nanjing 210023 China; ^5^ Institute of Science and Technology for Carbon Neutrality Southeast University Nanjing 210096 China; ^6^ Institute for Carbon Neutral Development Southeast University Nanjing Jiangsu 210096 China

**Keywords:** dual‐band regulation, electrochromic, radiative cooling, silver, solar heating

## Abstract

Electrochromic smart windows can actively modulate their reversible transition between transparent and opaque states, adapting to varying climatic conditions and thereby offering a sustainable solution for energy‐efficient buildings. However, the operational range of current electrochromic smart windows is mostly limited to the solar spectrum. Expanding this range into the mid‐infrared spectrum could significantly enhance their energy‐saving capabilities. In this study, a dynamic electrochromic (EC) glass that integrates silver electrodeposition/dissolution with mechanical flipping of the glass panel is designed. This design enables bidirectional dynamic modulation of both the solar spectrum (0.3–2.5 µm) and mid‐infrared spectrum (2.5–20 µm), with solar reflectance varying between 87.9% and 19.9%, and mid‐infrared emissivity varying between 90.6% and 10.8%. Consequently, the EC glass can dynamically switch between radiative cooling and solar heating modes. The simulation results show that the architectural application of this EC glass, with climate‐specific operating modes, can achieve a maximum of over 50% annual heating, ventilation, and air conditioning (HVAC) energy savings, contributing to carbon neutrality and sustainable development.

## Graphical abstract

In this study, an electrochromic glass is designed by effectively integrating silver electrodeposition with mechanical flipping of the glass panel, enabling bidirectional dynamic regulation of the solar and mid‐infrared spectra. Its capability to dynamically switch between radiative cooling and solar heating modes offers substantial energy‐saving potential for building applications.

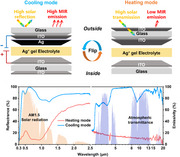

## 1 Introduction

Over the past three decades, global energy consumption has steadily increased due to enhanced electricity access in developing countries, rapid economic and population growth, and the frequent occurrence of extreme weather events. The buildings sector, which includes energy used for constructing, heating, cooling and lighting homes and businesses, as well as the appliances and equipment installed in them, accounts for over one third of global energy consumption and emissions.^[^ ^1^ ^]^ This presents significant environmental and economic challenges to sustainable development.^[^ ^2^, ^3^, ^4^ ^]^ Among these, heating, ventilation, and air conditioning (HVAC) systems consume over 40% of building energy, with projections indicating continued growth in building energy consumption over the next two decades.^[^ ^5^ ^]^ Windows, as the interface between indoor and outdoor environments, are a major source of energy loss, responsible for up to 60% of total building energy loss.^[^ ^6^ ^]^ Therefore, windows play a critical role in enhancing building energy efficiency. Traditional windows, as static components, cannot effectively respond to climate variations over time and space. In contrast, smart windows dynamically regulate the amount of solar radiation entering a building by reversibly switching between transparent and opaque states.^[^ ^7^ ^]^ This technology is pivotal in reducing HVAC energy consumption in buildings, while also enhancing visual comfort, providing natural daylighting, and reducing glare.^[^ ^8^, ^9^ ^]^

Electrochromic (EC), photochromic, thermochromic, and liquid crystal technologies are currently widely investigated in smart windows.^[^ ^10^, ^11^, ^12^, ^13^ ^]^ Among these, EC smart windows have garnered significant research attention due to their advantages, including active regulation, low energy consumption, fast response times, and a wide range of color changes. Electrochromism refers to the phenomenon in which certain materials undergo reversible changes in their electronic structure and optical properties—such as transmission, reflection, or absorption—when subjected to current or voltage, resulting in adjustable color or transparency. In recent decades, extensive research has focused on the transmission, reflection and color changes of traditional EC smart windows within the solar radiation spectrum.^[^ ^14^, ^15^, ^16^, ^17^, ^18^, ^19^, ^20^ ^]^ Among them, electrochromic devices based on reversible metal electrodeposition provide significant optical modulation and fast switching speed, enabling precise control over solar reflectance.^[^ ^21^, ^22^, ^23^, ^24^ ^]^ Building upon this foundation, it is critical to extend the modulation range of smart windows to the mid‐infrared (MIR) spectrum to dynamically control the radiative heat exchange between the building interior, the windows, and the exterior environment.^[^ ^25^, ^26^, ^27^, ^28^, ^29^ ^]^ Some works have reported outstanding MIR modulation performance through reversible metal electrodeposition.^[^ ^30^, ^31^ ^]^ However, the deposited states of metals such as silver (Ag) and copper (Cu) often correspond to high MIR reflectance, which is contrary to the requirement of high MIR emissivity for radiative cooling.^[^ ^32^, ^33^, ^34^, ^35^ ^]^ Therefore, it is essential to further explore and develop innovative EC structures based on reversible metal electrodeposition, which can dynamically regulate both solar and MIR radiation. In such structures, the deposited state corresponds to high solar reflectance and high MIR emissivity, enabling radiative cooling; whereas the transparent state corresponds to high solar transmittance and low MIR emissivity, allowing for solar heating. This dual‐mode regulation offers significant potential for achieving superior energy efficiency in buildings.

This study presents an EC glass designed to reduce building energy consumption. By effectively combining the Ag electrodeposition/dissolution with the mechanical flipping of the glass panel, the EC glass achieves bidirectional dynamic modulation over its optical properties in both the solar and MIR spectrum (Solar reflectance: 87.9% ⇌ 19.9%, MIR emissivity: 90.6% ⇌ 10.8%). This dynamic switching capability enables the EC glass to seamlessly transition between radiative cooling and solar heating modes, allowing it to adapt to varying climate conditions. With customizable operational modes, the proposed EC glass can be an ideal choice for smart windows worldwide.

## 2 Results and Discussion

### 2.1 Design Principles of the EC Glass

**Figure 1:**
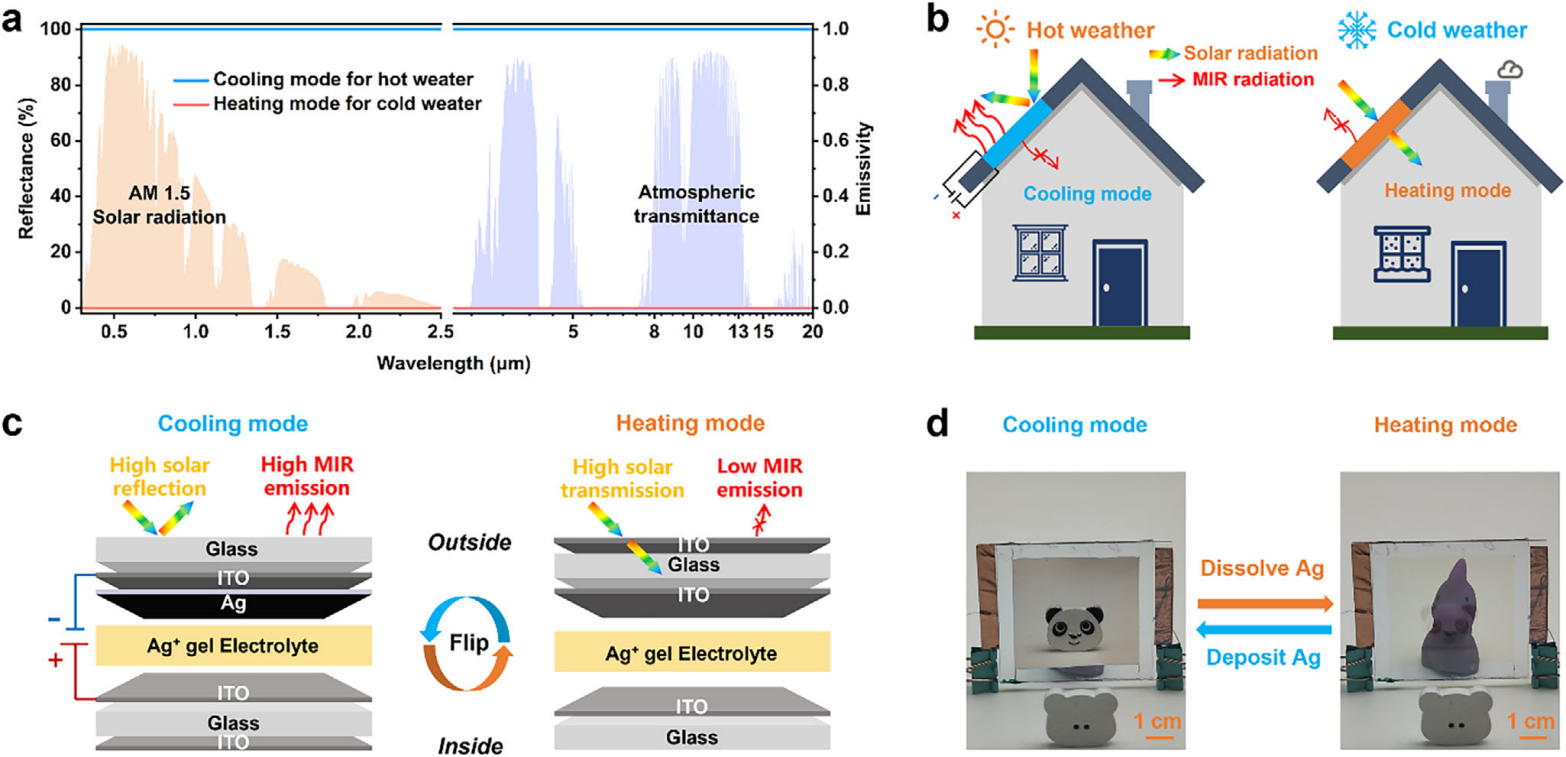
Structure and dual‐function modes of the EC glass. a) Optical properties of an ideal energy‐saving window in hot and cold weather. b) Schematic diagram of energy transfer for an ideal energy‐efficient skylight in hot and cold weather. c) EC glass structure with cooling mode (radiative cooling) and heating mode (solar heating) that can be dynamically switched. d) Photos of the EC glass in cooling mode and heating mode.

To minimize building energy consumption, energy‐efficient windows must address thermal regulation needs across both hot and cold weather. As shown in **Figure** **1**a,b, during the hot weather, solar radiation entering through the window can cause indoor overheating and glare.^[^ ^36^ ^]^ While visible light (0.4–0.76 µm) provides natural lighting and reduces the need for artificial illumination, studies have demonstrated that reflecting all solar radiation outdoors—rather than using spectral‐selective windows that transmit visible light while reflecting near‐infrared radiation—yields higher energy efficiency.^[^ ^37^ ^]^ Therefore, an ideal energy‐efficient window should exhibit near 100% solar radiation reflectance. Additionally, integrating MIR radiative cooling technology can further reduce the building's cooling load by emitting infrared radiation to the cold universe at a temperature of 3 K. The external surface of the energy‐efficient window should have high MIR emissivity to efficiently radiate indoor heat to the external environment, while the internal surface should have low MIR emissivity to minimize heat transfer into the interior. Conversely, during the cold weather, energy‐efficient windows should maximize the use of solar radiation for heating. In this case, the ideal window should exhibit 100% solar radiation transmittance to capture and utilize as much solar energy as possible, thereby reducing heating loads. To minimize radiative heat transfer between the window and the external environment, the external surface of the window should have low MIR emissivity to prevent heat loss to the cold exterior. Thus, the design of the EC glass should dynamically adjust its solar radiation reflectance and MIR emissivity in response to weather changes, optimizing energy utilization and thermal management performance.

### 2.2 Structure of the EC Glass

Based on reversible metal electrodeposition technology, we have designed a dual‐mode dynamic EC glass that integrates the electrodeposition/dissolution of Ag with the mechanical flipping of the glass panel. This design enables dynamic modulation of both the solar and MIR spectrum. The structure of the EC glass, as illustrated in Figure 1c, consists of two Indium Tin Oxide (ITO)‐coated glass electrodes enclosing an Ag‐based gel electrolyte. One electrode is single‐sided ITO‐coated glass, while the other is double‐sided ITO‐coated glass, creating a significant difference in emissivity between the inner and outer surfaces of the EC glass in the MIR spectrum. The thickness of the ITO layer is carefully optimized to achieve high transmittance in the solar spectrum while maintaining low emissivity in the MIR spectrum. During the hot weather, a negative voltage is applied to deposit Ag onto the ITO electrode surface to form a film, placing the EC glass in a high solar reflectance state. In this state, the outer surface of the EC glass is glass (soda‐lime glass) with high MIR emissivity, while the inner surface, coated with ITO, has low MIR emissivity. The high emissivity of the glass, coupled with the high solar reflectivity of the Ag film, facilitates radiative cooling. Simultaneously, the low emissivity ITO surface facing the interior reduces heat transfer from the external environment to the indoor space. In the cold weather, the EC glass undergoes a mechanical flip, and the applied voltage is removed, causing the Ag film to dissolve and alter its optical properties. At this point, the EC glass transitions to a high solar transmittance state, optimizing solar energy utilization for indoor heating. The ITO surface facing the exterior is characterized by low MIR emissivity, reducing the loss of indoor heat to the external environment. In summary, by combining electrochromic and mechanical flipping technologies, this design achieves bidirectional dynamic modulation of both high solar reflectance/high solar transmittance and high MIR emissivity/low MIR emissivity. This enables seamless switching between cooling mode (radiative cooling) and heating mode (solar heating). Moreover, the mechanical flipping function can be automated via power supply and programming, with a high degree of integration between the flipping mechanism and the electrochromic function, ensuring the overall effectiveness and efficiency of the system (Note S8, Supporting Information).

### 2.3 Preparation and Working Principle of the EC Glass

The EC glass forms a metallic Ag nanoparticle film through the electrochemical reduction of Ag ions, providing a high‐reflectivity surface. The preparation process is outlined in Figure S1 (Supporting Information). Initially, the ITO electrode undergoes surface treatment with oxygen plasma to remove residual organic impurities and hydroxylate the surface. A self‐assembled monolayer of 3‐mercaptopropyltrimethoxysilane (MPTMS) is then applied to the surface of the ITO electrode for modification. The trimethoxysilane groups anchor to the hydroxyl groups on the ITO electrode surface, while the terminal thiol groups interact strongly with the deposited Ag metal, ensuring the stability and high reflectivity of the Ag film (Note S1, Supporting Information).^[^ ^38^, ^39^ ^]^ Next, the Ag‐based gel electrolyte is encapsulated between two transparent ITO‐coated glass electrodes using VHB transparent adhesive, completing the assembly of the EC glass. The gel electrolyte is prepared by dissolving tetrabutylammonium bromide (TBABr), silver nitrate (AgNO₃), copper chloride (CuCl₂), and polyvinyl butyral (PVB) in dimethyl sulfoxide (DMSO).^[^ ^40^ ^]^ The formation and dissolution of the Ag film are governed by the electrochemical reduction of Ag⁺ and the mediated oxidation reactions in the electrolyte solution. Pulsed voltage can instantly apply a higher current density to the electrolyte thin layer near the electrode surface, thereby providing a smoother deposited film.^[^ ^41^ ^]^ When a −2.5 V pulse voltage is applied to the EC glass, Ag⁺ ions undergo electrochemical reduction and deposit as Ag nanoparticles on the ITO electrode (working electrode). At this potential, trace amounts of Cu^2^⁺ ions are also reduced, and the co‐deposition of Ag and Cu significantly enhances the uniformity of electrodeposition and improves the quality of the deposited film.^[^ ^40^, ^42^ ^]^ The deposited film consists of densely packed nanoparticles with diameters in the tens of nanometers, which facilitates the formation of a continuous and smooth layer, laying the foundation for high reflectivity (Note S2, Supporting Information). Concurrently, other ions on the counter electrode (such as Br⁻) undergo oxidation reactions to compensate for the reduction process. At this point, as shown in the left image of Figure 1d, the EC glass reflects a panda image positioned in front of it. After the removal of the applied voltage, due to the higher oxidation potential of Cu^+^ to Cu^2+^ compared to that of Ag to Ag⁺, Cu^2+^ mediates the oxidative dissolution of the deposited Ag film into Ag^+^ in the presence of Br⁻. This restores the EC glass to its initial high‐transparency state. In this state, as shown in the right image of Figure 1d, the purple ornament on the back of the EC glass becomes clearly visible. Additionally, cyclic voltammetry (CV) tests on the EC glass reveal clear reduction and oxidation behaviors of the Ag ions (Figure S5, Supporting Information).

The mechanism of silver deposition can be explained by the reduction reactions of silver halides, as shown in Equations (1) and (2):^[^ ^40^, ^43^ ^]^

(1) $$Ag^{+}+nBr^{-}\to AgBr_{n}^{1-n}$$

(2) $$AgBr_{n}^{1-n}+e^{-}\to Ag+nBr^{-}$$

At this stage, the oxidation reaction at the counter electrode is represented by Equation (3).

(3) $$3Br^{-}\to Br_{3}^{-}+2e^{-}$$

The oxidation of Ag is mediated by Cu ions in the presence of halide ions (such as Br⁻), as shown in Equation (4).

(4) $$Ag+Cu^{2+}+nBr^{-}\to AgBr_{n}^{1-n}+Cu^{+}$$

### 2.4 Optical Properties of the EC Glass

**Figure 2:**
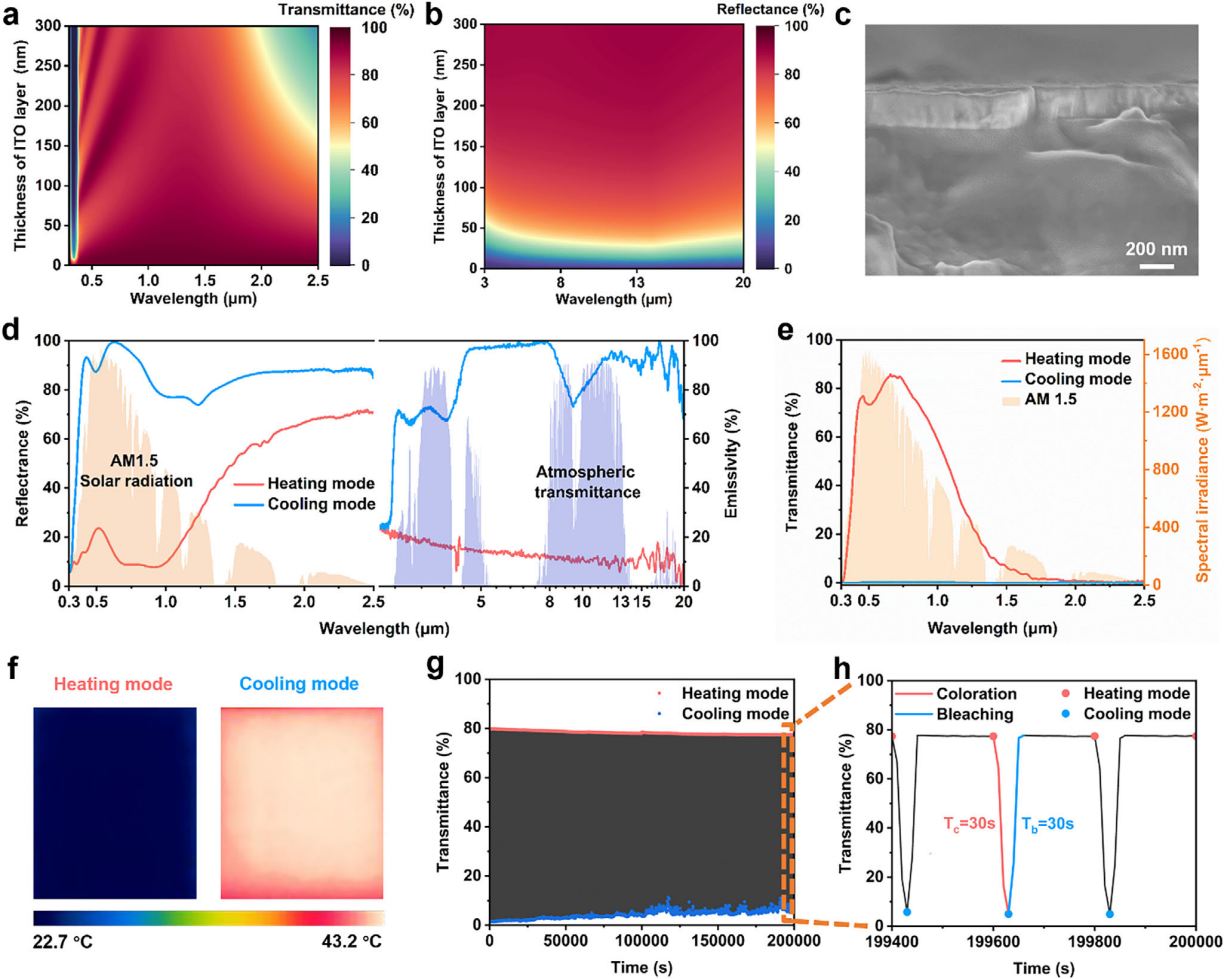
Spectral modulation capability and electrochemical properties of the EC glass. a) The relationship between solar transmittance and ITO film thickness. b) The relationship between MIR reflectance and ITO film thickness. c) Cross‐section of ITO‐coated glass captured by a SEM, showing the ITO film thickness. d) Comparison of solar reflectance and MIR emissivity of the EC glass in heating and cooling modes. e) Comparison of solar transmittance of the EC glass in heating and cooling modes. f) Infrared thermal imaging of the EC glass in heating and cooling modes measured on a thermal platform. g) The coloration‐bleaching cycling stability of the EC glass at 633 nm was evaluated following a sequence of −2.5 V (30 s) and 0 V (170 s) over a duration of 200 000 s. Considering that the maximum allowable measurement duration of the UV‐visible‐near infrared spectrophotometer is 100 000 s, two consecutive tests were performed accordingly. h) The last 600 s of the coloration‐bleaching cycle show the response of the optical properties of the EC glass to voltage.

The optimization of the ITO film thickness is one of the key factors in device design. An optimal thickness allows the ITO film to maintain high solar transmittance while achieving high MIR reflectance. The relationships between ITO film thickness and solar transmittance, as well as MIR reflectance, were calculated using the transmission matrix method, with the specific results shown in **Figure** **2**a,b. The refractive index of ITO was obtained by the dielectric constant, which was calculated using the Drude model:^[^ ^44^ ^]^

(5) $$\varepsilon \;\;\left(\omega \right)=\varepsilon_{\infty }-\frac{\omega_{p}^{2}}{\omega \left(\omega -i\gamma \right)}$$

where ε_∞_ = 3.95, ω_*p*_ = 3.067 × 1015 rad s^−1^, and γ = 1.822 × 10^14^ Hz. They are represented as the relative permittivity at infinite frequency, plasma frequency, and collision frequency of ITO, respectively. From Figure 2a,b, it can be observed that as the ITO film thickness increases, solar transmittance gradually decreases (due to reduced near‐infrared transmittance, while visible light remains largely unchanged), while MIR reflectance gradually increases (reaching a maximum of ≈89%, approaching its highest level at ≈190 nm, after which the rate of increase significantly slows down). Therefore, we selected an ITO film with a thickness of 190 nm to achieve high solar transmittance (particularly in the visible range) and low MIR emissivity (with nearly zero MIR transmittance of ITO). In addition, the Ultraviolet (UV) transmittance of glass coated with a 190 nm‐thick ITO layer is 53.6%, compared to 81.0% for bare glass, which imparts a certain degree of UV‐aging resistance to the EC glass (Note S3, Supporting Information). The scanning electron microscope (SEM) cross‐section shown in Figure 2c clearly reveals the thickness and morphology of the ITO film. Further confirmation of the ITO film's thickness was obtained through Energy Dispersive Spectroscopy Line Scan analysis (Figure S6, Supporting Information). Combining the SEM surface morphology of the ITO film shown in Figure S7 (Supporting Information) and the X‐ray diffraction analysis presented in Figure S8 (Supporting Information), it is evident that the film exhibits a face‐centered cubic structure, good crystallinity, and distinct characteristic diffraction peaks.

The EC glass, constructed with this ITO film, demonstrates bidirectional dynamic modulation in both the solar and MIR spectrum, as illustrated in Figure 2d,e. The average solar reflectance and MIR emissivity of the EC glass were calculated by weighting according to the solar radiation intensity (AM 1.5) and blackbody radiation intensity (300 K), respectively (Note S4, Supporting Information).^[^ ^45^ ^]^ When a −2.5 V pulse voltage is applied, Ag deposition results in an average reflectance of 87.9% (with an average transmittance of 0.09%) in the solar spectrum, effectively blocking external heat from entering the interior. In this state, the outward‐facing glass exhibits an average emissivity of 90.6% in the MIR range (with an average emissivity of 87.1% in the 8–13 µm atmospheric window), efficiently radiating indoor heat to the external environment. Combined with the high reflectance of solar radiation, this state enables the EC glass to achieve radiative cooling. Upon removal of the voltage, the Ag dissolves through a mediated process, and the EC glass returns to its initial transparent state. In the solar spectrum, the EC glass has an average transmittance of 61.2% (with an average reflectance of 19.9%), allowing for effective collection and utilization of solar energy for heating. Notably, the EC glass exhibits an average transmittance of 78.8% in the visible spectrum, ensuring sufficient daylighting. We captured color images of the EC glass under uniform backlighting and converted them into the Lab color space for analysis, revealing excellent visible clarity and color uniformity (Note S5, Supporting Information).^[^ ^46^ ^]^ At this point, the outward‐facing surface is the ITO film, which has an average emissivity of 10.8% in the MIR spectrum (with an average emissivity of 10.5% in the 8–13 µm atmospheric window), effectively reducing heat loss from the interior to the exterior. The EC glass thus operates in solar heating mode. To further validate the emissivity differences in the MIR spectrum resulting from the dual‐sided flipping of the EC glass, two identical pieces of EC glass were placed on the same heating plate (set to 50 °C), with the glass and ITO film facing upward, respectively (Figure 2f). After temperature stabilization, significant differences were observed using a thermal imaging camera. The glass side exhibited high emissivity in the MIR spectrum, resulting in an increased apparent temperature. In contrast, the ITO film side displayed a lower apparent temperature due to its low emissivity in the MIR spectrum.

### 2.5 Electrochromic Performance of the EC Glass

Coloration efficiency (CE) is an important parameter to assess the electrochromic performance of devices. The calculation method is detailed in Note S7 (Supporting Information). By applying a constant voltage of −2.5 V and simultaneously monitoring the transmittance at 633 nm, we obtained a CE of 118.1 cm^2^/C for the EC glass (Figure S16, Supporting Information), indicating fast response and low energy consumption.^[^ ^47^ ^]^ To evaluate the stability of the EC glass, we first examined its optical properties under a constant applied voltage. By continuously applying a −2.5 V pulse voltage and recording its optical characteristics at a wavelength of 633 nm, Figure S17 (Supporting Information) shows that the solar reflectance of the EC glass at 633 nm remained ≈98% over a period of 100 000 s, indicating its excellent potential for long‐term stable operation. Subsequently, we applied a periodic voltage switching protocol (−2.5 V for 30 s → 0 V for 170 s) to the EC glass and monitored the in‐situ transmittance at 633 nm to evaluate its switching ability between the radiative cooling and solar heating modes. As shown in Figure 2g, after 200 000 s and 1000 coloration‐bleaching cycles, the EC glass maintained 92.5% of its initial transmittance modulation, exhibiting excellent cycling stability without obvious degradation. The cycled EC glass still maintains good optical clarity and color uniformity (Note S5, Supporting Information), attributed to its preserved electrochemical reversibility after coloration‐bleaching cycles, as evidenced by the CV curves of the cycled EC glass (Figure S18, Supporting Information), which continue to exhibit pronounced redox peaks associated with Ag deposition/dissolution. We further analyzed the optical response over the last 600 s of the cycling process (Figure 2h), where the EC glass demonstrated rapid response to voltage changes: the transmittance at 633 nm decreased rapidly from the initial 77.4–4.9% within 30 s after applying the voltage, and recovered back to its initial value within 30 s after the voltage was removed. Additionally, we analyzed the optical response during the initial 600 s (Figure S19, Supporting Information), showing that at the beginning of operation, the coloration time was 30 s (transmittance changing from 79.8% to 1.4%), and the bleaching time was 90 s. A similar trend is also observed in the corresponding current–time curves (Figures S20–S22, Supporting Information), indicating a progressive activation of the EC glass during continuous switching. In the initial cycles, the interaction between the thiol self‐assembled monolayer and the ITO surface may not be fully optimized. Repeated Ag deposition/dissolution promotes the formation of more stable coordination bonds between thiol groups and Ag nanoparticles and facilitates electrochemical cleaning of residual surface impurities, exposing more active sites and enhancing interfacial charge transfer efficiency (see Figure S23, Supporting Information, for the coloration‐bleaching behavior of the uncleaned/unmodified EC glass). In summary, the EC glass demonstrates good long‐term stability, rapid response, and low degradation during dynamic electrochromic regulation, exhibiting efficient switching capabilities between radiative cooling and solar heating modes.

### 2.6 Outdoor Thermal Measurement of the EC Glass

**Figure 3:**
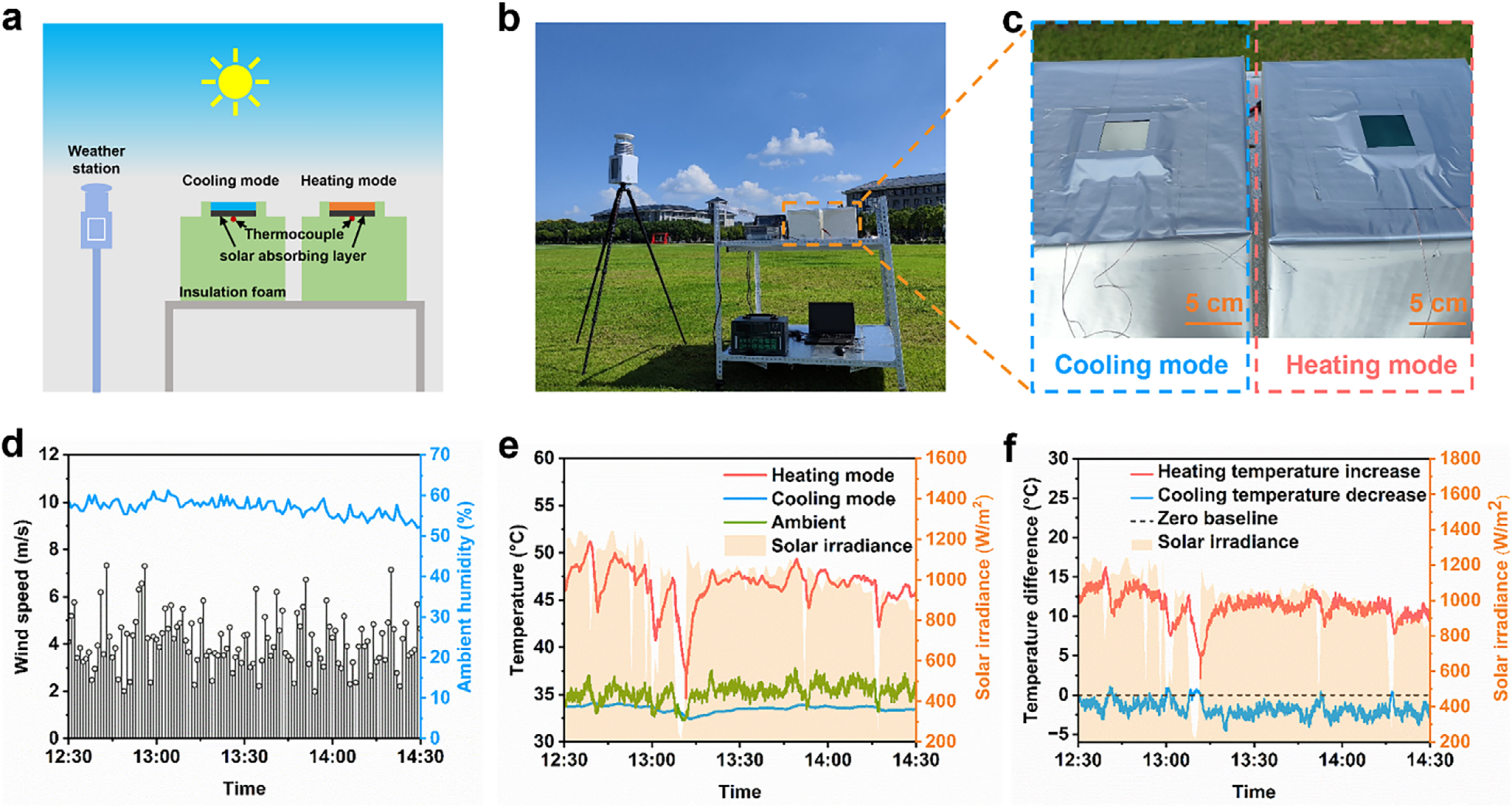
Outdoor experimental setup and measured thermal performance of the EC glass. a) Schematic diagram of outdoor experimental setup. b) Photo of outdoor experimental setup and weather. c) Photos of the EC glass in cooling and heating modes during outdoor experiment. d) Wind speed and ambient humidity during the outdoor experiment. e) Temperature of the solar absorbing layer of the EC glass under direct sunlight in heating and cooling modes. f) Temperature difference between the solar absorbing layer on the back of the EC glass and the surrounding environment during the outdoor experiment.

To assess the temperature management capabilities of the EC glass in an outdoor environment, we designed and constructed an outdoor experimental setup, as shown in **Figure** **3**a. To enable direct comparison, two identical EC glass samples were mounted on a plane facing the sky. Given that the initial state of the EC glass exhibits high solar transmittance, we added a black solar absorbing layer to the back of the setup, which has an average absorption rate of 97.4% across the solar spectrum (Figure S24, Supporting Information). This layer was used to convert all solar radiation passing through the EC glass into heat, preventing measurement errors caused by direct sunlight. Thermocouples were tightly attached to the back of the solar absorbing layer to accurately measure the temperature. Additionally, to minimize thermal losses between the system and the environment, the outer layer of the setup was constructed with foam polystyrene material and wrapped in aluminum foil, which helped reduce the impact of direct solar heating on the measurements. Figure S25 (Supporting Information) shows a digital photograph of the outdoor experimental setup, including the experimental group, data acquisition equipment, waveform generator, Alternating Current power supply, and testing computer.

The outdoor experiment took place on September 18, 2024, at noon. The weather conditions on the outdoor experimental day were relatively clear, with few clouds, and a specific sky photo is shown in Figure S26 (Supporting Information). The outdoor experiment was conducted at Southeast University's Jiulong Lake campus in Nanjing, China (31.88°N, 118.82°E, 30 m elevation), on an open grass field. Figure 3b presents a digital photograph of the outdoor experiment, showing the outdoor experimental setup and meteorological station, providing an overview of the surrounding environment. Figure 3c illustrates the specific states of the EC glass under radiative cooling mode and solar heating mode during the outdoor experiment. In the outdoor experiment, one EC glass sample had its glass surface facing the sky, with a −2.5 V pulse voltage applied to induce Ag deposition, thereby activating the radiative cooling mode. The other EC glass sample, with its ITO surface facing the sky, remained in its initial state without any applied voltage, maintaining the solar heating mode. Data on wind speed, relative humidity, and solar irradiance are shown in Figure 3d,e, respectively. Due to environmental factors such as cloud distribution, wind speed variations, and fluctuations in solar irradiance, the temperature measurements exhibited some fluctuations within a certain range. Figure 3e presents the detailed temperature test results. Under radiative cooling mode, the temperature of the solar absorbing layer at the back was lower than the ambient temperature. Conversely, in solar heating mode, the temperature of the solar absorbing layer at the back was significantly higher than the ambient temperature. To present the temperature measurement results more clearly, Figure 3f shows the temperature differences between the solar absorbing layer at the back and the ambient temperature under both modes. The outdoor experimental results demonstrate that under ≈980 W m^−2^ of direct solar radiation, in radiative cooling mode, the temperature of the solar absorbing layer at the back of the EC glass was, on average, 1.7 °C lower than the ambient temperature, with a maximum temperature difference of 4.5 °C. In solar heating mode, the temperature of the solar absorbing layer at the back was, on average, 11.3 °C higher than the ambient temperature, with a maximum temperature difference of 16.2 °C. In conclusion, the EC glass exhibited excellent temperature management capabilities in the real outdoor environment, demonstrating exceptional performance in both radiative cooling and solar heating modes.

### 2.7 Architectural Applications and Energy Efficiency Analysis of the EC Glass

The dual‐mode EC glass is highly versatile and can be applied across a wide range of architectural settings, including skylights, transparent roofs, glass atria, and other transparent building envelope structures in environments such as malls, offices, airports, etc. It offers the potential to replace conventional glass, allowing for dynamic adjustment of its working modes based on the heating and cooling demands specific to different climates and regions, thereby improving building energy efficiency. In cooling scenarios, a −2.5 V pulse voltage is applied with the glass surface facing outward, activating the radiative cooling mode, which features high solar reflectance and high MIR emissivity. Conversely, when heating is necessary, the ITO surface is oriented outward, and the voltage is removed, shifting the EC glass into solar heating mode with high solar transmittance and low MIR emissivity. Since both electrochromic and mechanical actuation require power, an automated control system can be employed to manage the state of the EC glass, further optimizing its energy‐saving performance (Note S8, Supporting Information).

**Figure 4:**
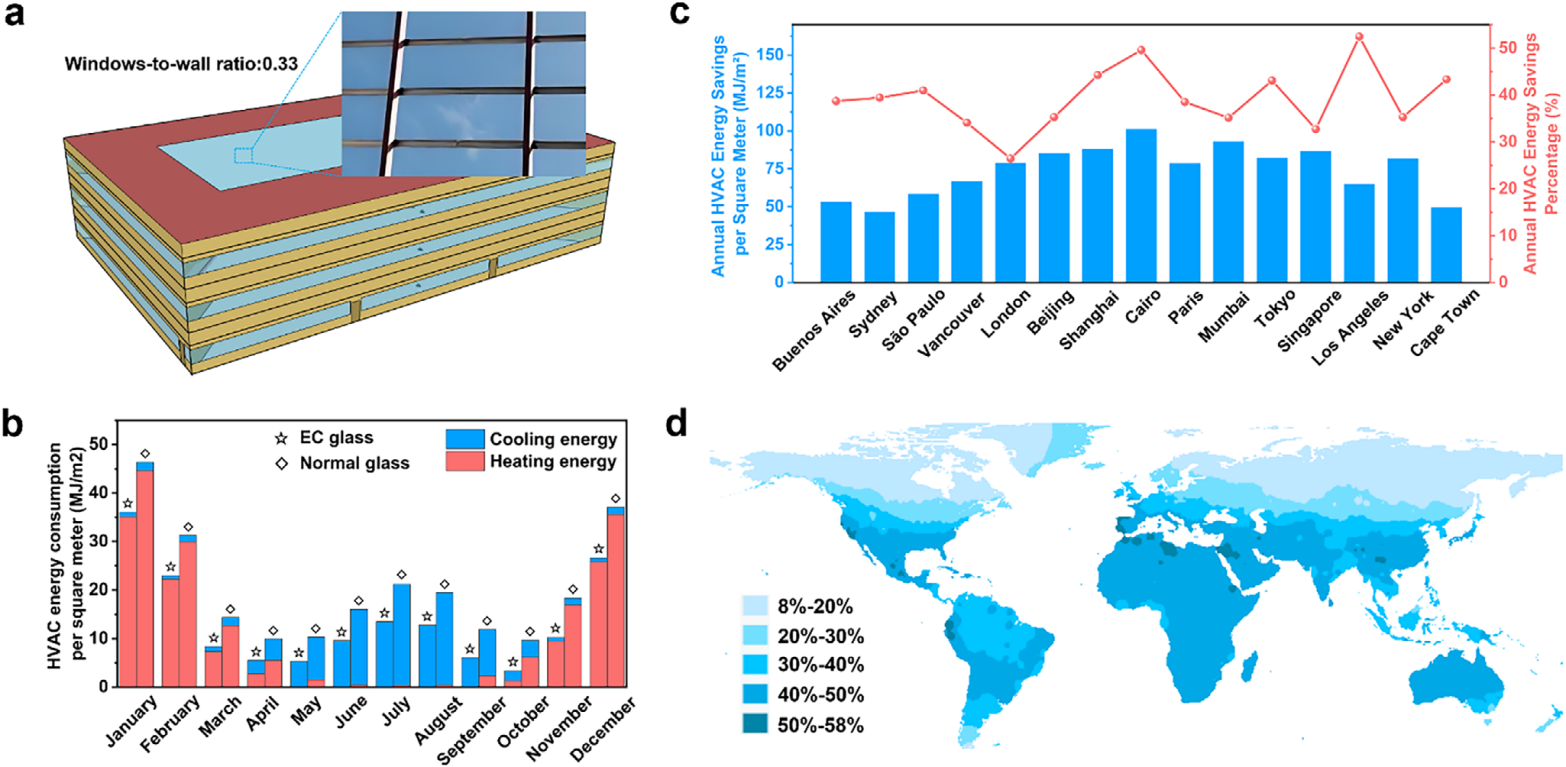
HVAC energy‐saving effects of the EC glass applied to buildings. a) Schematic illustration of the model where the EC glass is applied to building skylights. b) Under Beijing's climate conditions, the cooling and heating energy consumption achieved by applying the EC glass to building skylights over the 12 months of a year (in MJ/m^2^). c) Fifteen representative cities from different climate zones were selected to evaluate the energy‐saving effects of applying the EC glass to building skylights (comparison of annual HVAC energy savings per square meter, MJ/m^2^, and annual HVAC energy savings percentage, %). d) Considering the local climate conditions, the annual HVAC energy savings percentage resulting from the global application of the EC glass.

To evaluate the energy‐saving performance of the EC glass in buildings, we utilized Energy Plus software to simulate the annual energy savings in HVAC systems when EC glass is applied to commercial building skylights. The analysis utilized a prototype medium‐sized office building model from the U.S. Department of Energy, designed in accordance with the ASHRAE 90.1 standard (see Table S1, Supporting Information, for details).^[^ ^48^, ^49^ ^]^ The building features a window‐to‐wall ratio of 33%, with a total floor area of 4982.19 square meters, and skylights positioned on the roof, preserving the same 33% window‐to‐wall ratio (as shown in **Figure** **4**a). For the simulation, we first used normal glass as the skylight, establishing it as the baseline for HVAC energy consumption (see Table S2, Supporting Information, for details). We then assessed potential HVAC energy savings by dynamically adjusting the skylight's optical properties based on the developed EC glass's performance in both radiative cooling and solar heating modes (see Table S3, Supporting Information, for details). To simplify the analysis, the energy consumption during the coloring process was neglected (assuming 180 cooling days per year, five color‐switching cycles per day, with each coloring lasting 30 s; the energy consumption for coloring accounted for less than 1% of the annual HVAC energy savings in a typical scenario such as Beijing). It is worth mentioning that the energy needed to sustain the radiative cooling state is minimal. After the coloring process is finished, the electrolyte's resistance increases significantly (for instance, the resistance of a 5 cm × 5 cm panel is ≈10 MΩ).^[^ ^22^ ^]^ Dynamic switching conditions for the skylights were configured in EnergyPlus, with the EC skylights set to radiative cooling mode when the temperature exceeded 20 °C, and to solar heating mode when the temperature fell below 20 °C.

To begin, we selected Beijing, a city with a typical summer‐hot, winter‐cold climate, for an analysis of building HVAC energy consumption across different months of the year. The simulation results, shown in Figure 4b, indicate that ☆ represents the monthly HVAC energy consumption for buildings with EC glass skylights, while ◇ represents the monthly HVAC energy consumption for buildings with normal glass skylights. The data clearly demonstrates that, compared to the normal glass, the EC glass offers energy‐saving benefits throughout all 12 months. Additionally, we selected 15 representative cities from various climate zones for an annual HVAC energy‐saving analysis. The results, shown in Figure 4c, reveal that the annual HVAC energy savings exceeded 25% in these cities, with the majority achieving savings greater than 50 MJ/m^2^. Detailed results are provided in Table S4 (Supporting Information). To further validate the energy‐saving potential of the EC glass, we performed an extensive analysis of HVAC energy savings in over 1000 cities worldwide, as illustrated in Figure 4d. The findings show that the application of the EC glass in building skylights demonstrates significant energy‐saving potential globally, with a maximum of over 50% annual HVAC energy savings (specific value distribution shown in Figure S28, Supporting Information). More specifically, in the majority of regions worldwide, the annual HVAC energy savings exceed 50 MJ m^−2^, with some regions achieving annual HVAC energy savings of over 150 MJ m^−2^ (Figures S29 and S30, Supporting Information).

## 3 Conclusion

To minimize building energy consumption, it is crucial to consider both thermal regulation technologies for hot and cold weather, optimizing the use of dual‐band solar and MIR radiation for smart windows. In this context, we have developed a dual‐mode dynamic EC glass based on reversible metal electrodeposition technology. This EC glass integrates electrically driven Ag deposition/dissolution with mechanically driven flipping of the glass panel, enabling bidirectional dynamic modulation of both the solar spectrum and the MIR spectrum. In hot weather, applying voltage induces Ag deposition, orienting the glass surface outward and achieving 87.9% solar reflectance and 90.6% MIR emissivity. This configuration activates the radiative cooling mode, enhancing cooling performance. In cold weather, removing the voltage causes the Ag to dissolve and the ITO surface to face outward, resulting in 61.2% solar transmittance and 10.8% MIR emissivity. This transition initiates solar heating mode, allowing more solar radiation to penetrate the interior. The EC glass undergoes color change and bleaching in less than 30 s, and after 1000 cycles, its performance shows no significant degradation, demonstrating excellent stability and durability. Outdoor experiments reveal that under ≈980 W m^−2^ of direct solar radiation, the EC glass achieves sub‐environmental cooling, with its radiative cooling mode averaging 1.7 °C lower than ambient temperature, while solar heating mode averages 11.3 °C higher than the ambient temperature, highlighting its exceptional thermal management capability. Additionally, EnergyPlus simulations indicate that this EC glass, with customized operating modes for diverse global climates, can achieve a maximum of over 50% HVAC energy savings, making it a highly effective choice for smart windows in various climates worldwide.

## 4 Experimental Section

### Materials

AgNO₃ (Sigma–Aldrich Co. Inc.), TBABR (Sigma–Aldrich Co. Inc.), CuCl₂ (Aladdin Industrial Co. Ltd.), DMSO (Aladdin Industrial Co. Ltd.), PVB (Aladdin Industrial Co. Ltd.), MPTMS (Sigma–Aldrich Co. Inc.), and ITO glass (5 cm × 5 cm × 1.1 mm, Huanan Xiangcheng Tech Ltd.).

### MPTMS Monolayer Self‐Assembly

The ITO electrodes were sequentially cleaned ultrasonically in acetone, ethanol, isopropanol, and deionized water for 15 min each, followed by drying in a nitrogen atmosphere. The electrodes were then treated with oxygen plasma for 10 min. After plasma treatment, the ITO electrodes were immersed in a solution of 2.5% MPTMS in toluene (v/v) for 12 h. Subsequently, the electrodes were ultrasonically cleaned in ethanol for 15 min to remove any unbound monomers. Finally, the surface‐modified ITO electrodes were dried in a nitrogen atmosphere.

### Preparation of Electrolyte and EC Glass

In this study, AgNO₃ (0.5 mmol, 42.5 mg), CuCl₂ (0.097 mmol, 6.5 mg), TBABr (2.5 mmol, 403 mg), DMSO (5 mL), and PVB (10 wt%, 0.6 g) were mixed at 800 rpm for at least 12 h to form an electrolyte gel. The gel was then sandwiched between two surface‐modified ITO electrodes to create EC glass with dimensions of 5 cm × 5 cm. The electrolyte was sealed using 0.5 mm thick VHB transparent adhesive.

### Characterization

A waveform generator (Agilent, 33210A) was used to apply a pulse voltage. Partial electrochemical characterizations were conducted using an electrochemical workstation (CHI760E). The transmittance and reflectance of the EC glass in the 0.3–2.5 µm range were measured using a UV–visible–near infrared spectrometer (UV‐3600i Plus). The emissivity of the EC glass in the 2.5–20 µm range was measured using a Fourier‐transform infrared spectrometer (Nicolet iS50). The microstructure and elemental composition of the ITO film and the films deposited on the ITO electrode were characterized using a scanning electron microscope (Crossbeam 350, SEM450). The phase structure of the ITO was analyzed using an X‐ray diffractometer (D8). The elemental chemical states of the deposited films were analyzed using X‐ray photoelectron spectroscopy (Thermo Scientific K‐Alpha).

## Conflict of Interest

The authors declare no conflict of interest

## Supporting information



Supporting Information

## Data Availability

The data that support the findings of this study are available from the corresponding author upon reasonable request.
