# Supplementary material for: Dual‐Band Electrochromic Smart Window for Dynamic Switching Between Radiative Cooling and Solar Heating
Source: Adv Sci (Weinh). 2025 May 20;12(30):e04483. doi: 10.1002/advs.202504483 (PMC12376666; doi:10.1002/advs.202504483)
Supplement: Supplementary file 1 — Supporting Information [file ADVS-12-e04483-s001.docx]

Supporting information for：

**Dual-Band Electrochromic Smart Window for Dynamic Switching Between Radiative Cooling and Solar Heating**

Xinyu Zhao^1^, Qixiang Chen^1^, Fan Fan^1^, Qiliang Wang^2^, Yuehong Su^2^, Minsu Liu^3^, Fahua Zhu^4^, Dongliang Zhao^1,5,6*^

*1 School of Energy and Environment, Southeast University, Nanjing, Jiangsu 210096, China*

*2 Department of Architecture and Built Environment, University of Nottingham, Nottingham NG7 2RD, UK*

*3 ARC Research Hub for Smart Process Design and Control, Department of Chemical Engineering, Monash University, VIC 3800, Australia*

*4 State Key Laboratory of Low-carbon Smart Coal-fired Power Generation and Ultra-clean Emission; China Energy Science and Technology Research Institute Co.,Ltd., Nanjing 210023, China*

*5 Institute of Science and Technology for Carbon Neutrality, Southeast University, Nanjing 210096, China*

*6 Institute for Carbon Neutral Development, Southeast University, Nanjing, Jiangsu, 210096, China*

**Author to whom correspondence should be addressed. Email:* [*dongliang_zhao@seu.edu.cn*](mailto:dongliang_zhao@seu.edu.cn)

**
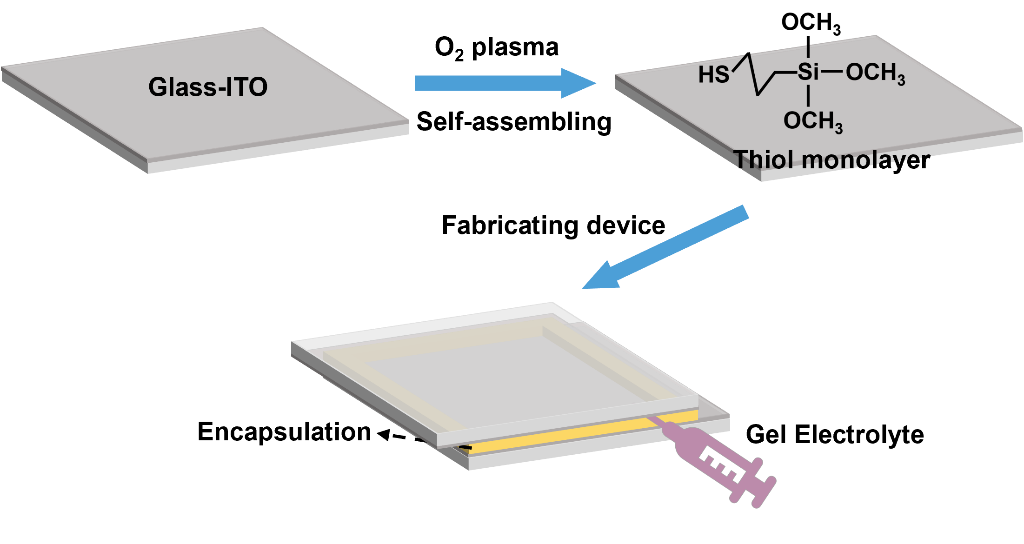
**

**Figure S1.** Schematic of the preparation process of the electrochromic (EC) glass.

**Note S1. Oxygen Plasma Cleaning and MPTMS Modification**

During the EC glass fabrication (as shown in Figure S1), we subjected the Indium Tin Oxide (ITO) electrodes to oxygen plasma treatment to remove organic residues and hydroxylate the surface. Subsequently, a self-assembled monolayer of 3-mercaptopropyltrimethoxysilane (MPTMS) was applied to the ITO surface. The trimethoxysilane groups anchor to the hydroxyl groups on the ITO surface, while the terminal thiol groups form strong interactions with the deposited silver (Ag), ensuring the deposited film’s stability and high reflectivity.

To evaluate the effect of plasma cleaning and MPTMS modification, we compared the electrochromic response and Ag deposition behavior of cleaned/modified versus uncleaned/unmodified ITO electrodes. As shown in Figure S2, the cleaned/modified EC glass achieved ~96% reflectance in a short time and maintained this level under a -2.5 V pulsed voltage, whereas the uncleaned/unmodified EC glass showed a sudden stagnation in reflectance increase. To investigate the cause, we retrieved and photographed both EC glasses after 10 minutes of voltage application. The corresponding deposition states are shown in the insets of Figure S2. The cleaned/modified EC glass displayed a stable, smooth mirror-like Ag film, whereas the uncleaned/unmodified EC glass showed a ruptured film. This can be attributed to the plasma treatment exposing more active sites and the strong interaction between the thiol groups and deposited Ag metal, resulting in a more uniform, stable, dense, and low-roughness Ag film.


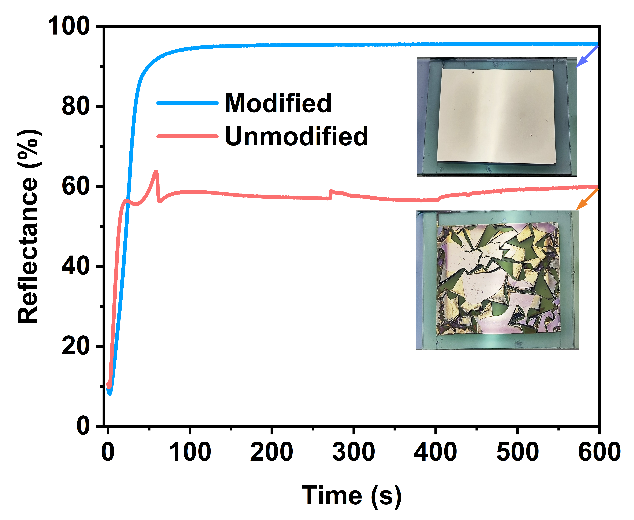


**Figure S2.** Reflectivity changes at a wavelength of 633 nm were measured under a -2.5 V pulsed voltage for cleaned/modified EC glass and uncleaned/unmodified EC glass. The inset shows the state of the deposited films after 10 minutes of voltage application.

**Note S2. Microstructure and Elemental Analysis of the Deposited Film**

We conducted a combined Scanning Electron Microscopy (SEM) and Energy Dispersive Spectroscopy (EDS) analysis of the film deposited for 300 seconds under a -2.5 V pulsed voltage, as shown in Figure S3. At low magnification (Figure S3a), a uniform and compact deposited film was observed. EDS mapping of the same region (Figure S3b) revealed that the film consists of co-deposited Ag and copper (Cu), with both elements distributed homogeneously. The atomic percentage of Cu was approximately 27.6% relative to Ag. Co-deposition of Ag and Cu significantly enhances the uniformity of the electrodeposition process and improves the quality of the film ^[1,2]^. At higher magnification (Figure S3c), the film was found to be composed of densely packed Ag nanoparticles, each with a diameter of several tens of nanometers, which facilitates the formation of a continuous and smooth film—critical for achieving high reflectivity.

Furthermore, we performed X-ray photoelectron spectroscopy (XPS) characterization on the film deposited for 300 seconds under a -2.5 V pulsed voltage to determine the chemical states of Ag and Cu, as shown in Fig S4. First, both Ag and Cu were simultaneously detected on the film, further confirming that the film was formed by the co-deposition of Ag and Cu. For the XPS results of Ag shown in Fig S4a, the Ag 3d orbital splits into two peaks, Ag 3d_5/2_ and Ag 3d_3/2_, corresponding to binding energies of 368.2 eV and 374.2 eV, respectively, which are consistent with the binding energies of metallic Ag. Additionally, the presence of two characteristic energy loss peaks and the relatively narrow full width at half maximum (FWHM) further confirm the existence of metallic Ag. For the XPS results of Cu shown in Fig S4b, the Cu 2p orbital splits into Cu 2p_3/2_ and Cu 2p_1/2_ peaks, corresponding to binding energies of 932.5 eV and 952.2 eV, respectively, matching the binding energies of metallic Cu. The relatively narrow FWHM further confirms the presence of metallic Cu.


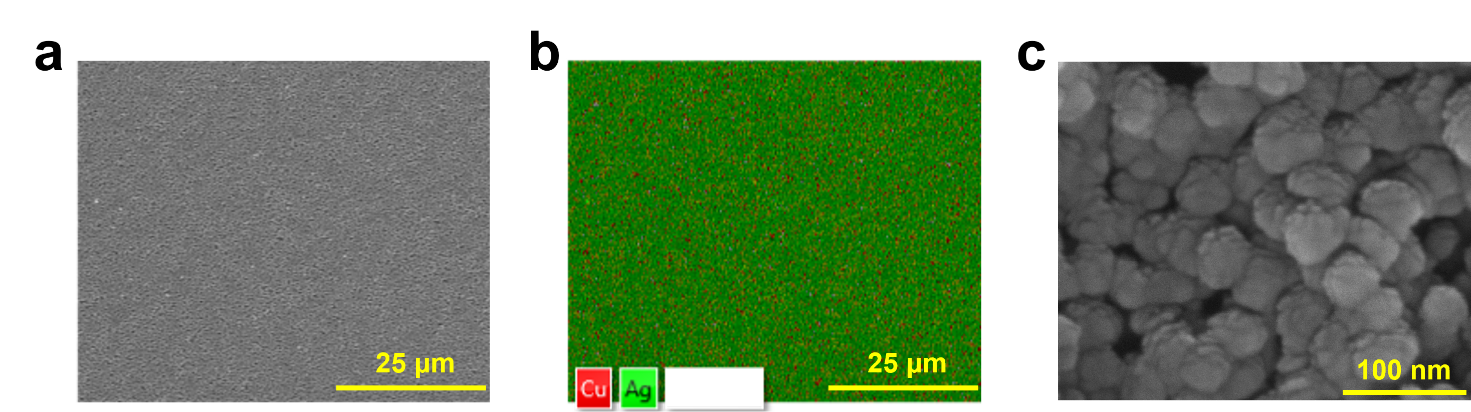


**Figure S3.** SEM and EDS characterization of the deposited film. (a) Low-magnification morphology of the deposited film. (b) EDS analysis of the region shown in (a). (c) High-magnification morphology of the deposited film.


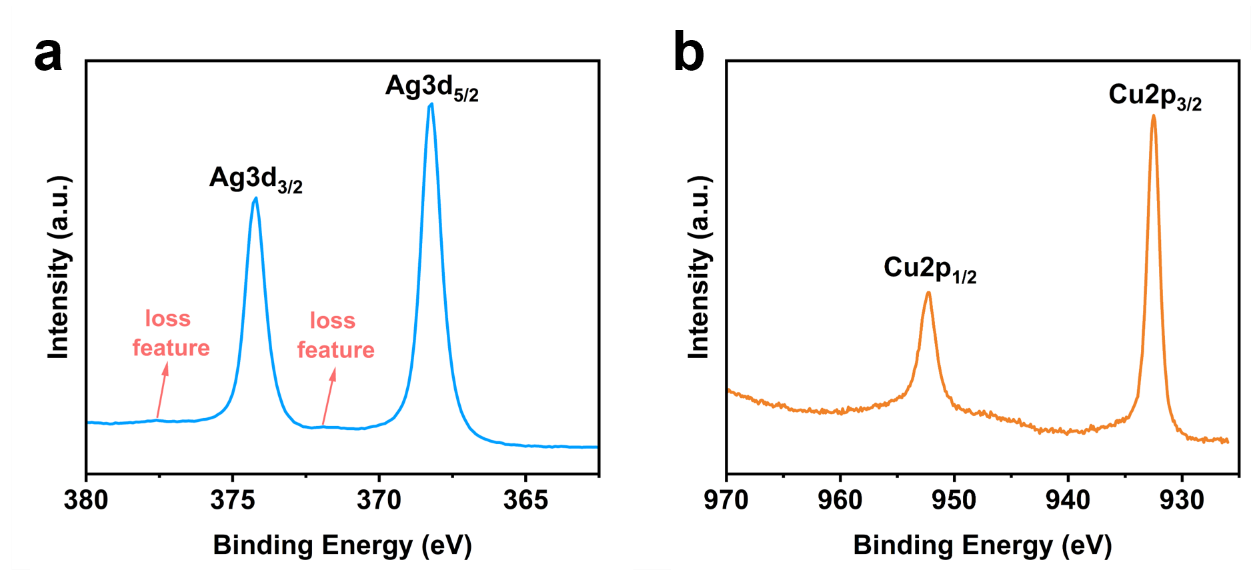


**Figure S4.** XPS characterization of the deposited film. (a) Ag 3d, (b) Cu 2p.

**
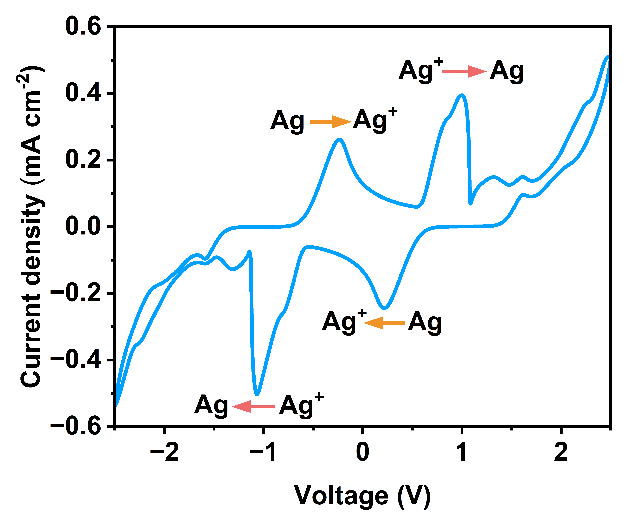
**

**Figure S5.** CV of the EC glass with a dual-electrode system, tested at a scan rate of 10 mV/s.

**
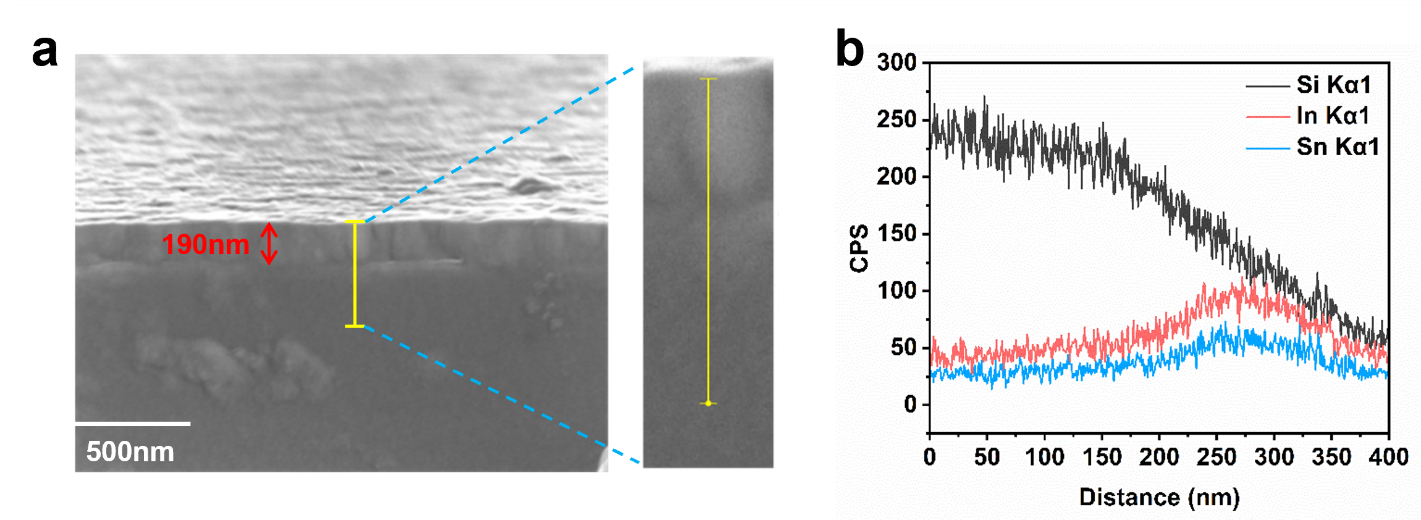
**

**Figure S6.** (a) SEM cross-sectional image of the ITO film and EDS line scan analysis. (b) Element-distance relationship obtained from the EDS line scan.


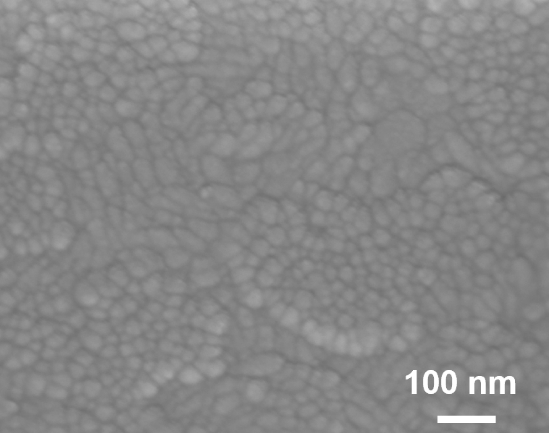


**Figure S7.** SEM surface image of the ITO film.


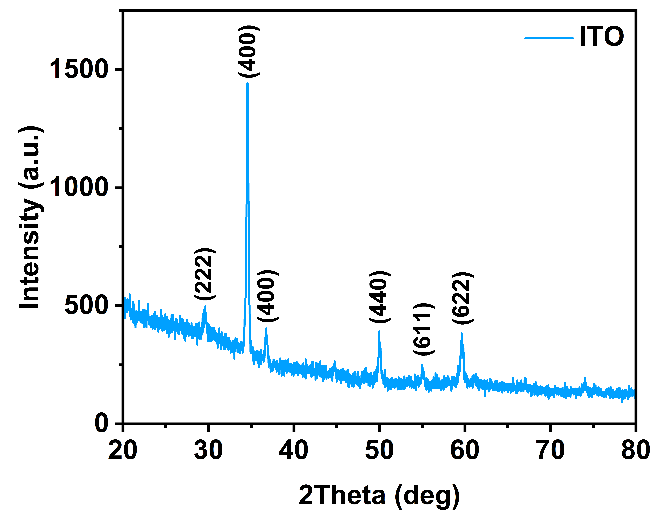


**Figure S8.** XRD pattern of the ITO film.

**Note S3. UV Stability of the EC Glass**

In practical applications, the environmental durability of the EC glass must account for factors such as Ultraviolet (UV)-induced Ag⁺ reduction and long-term electrolyte aging. In particular, Ag⁺ ions in gel-based electrolytes can undergo photoreduction under UV exposure, leading to the uncontrolled formation of Ag nanoparticles, which may result in loss of reversibility and deterioration of optical performance. To assess this, the EC glass was continuously exposed to UV light at an intensity of 500 mW/cm² for 7 days, and its transmittance was measured daily to monitor any decline in optical performance due to the formation of Ag nanoparticles. As shown in Figure S9, a slight decrease in visible light transmittance was observed after the first day, followed by a very slow decline over the subsequent days (the solid blue circles in the figure indicate the variation in transmittance at 550 nm from day 0 to day 7 under UV exposure). These results suggest that the EC glass possesses a certain degree of UV aging resistance. To further verify the electrochemical reversibility after UV aging, we measured the Cyclic voltammogram (CV) curve of the EC glass after 7 days of UV irradiation, as presented in Figure S10. Pronounced oxidation and reduction peaks of Ag were still observed, indicating that the device maintained good reversibility of Ag deposition/dissolution even after accelerated UV aging.

Additionally, we investigated the UV optical properties of ITO-coated glass and normal glass to analyze the potential reason for the enhanced UV resistance of the EC glass. As shown in Figure S11, the glass coated with a 190 nm-thick ITO layer exhibited a lower UV transmittance compared to normal glass. Specifically, the average AM1.5-weighted transmittance of the ITO-coated glass in the 0.3-0.4 μm wavelength range was 53.6%, whereas that of normal glass was 81.0% (wavelengths below 0.3 μm are largely absorbed by the atmosphere and ozone layer and were not considered). This finding indicates that the ITO-coated glass can partially attenuate UV irradiation reaching the electrolyte, thereby enhancing the UV aging resistance of the EC glass.

**
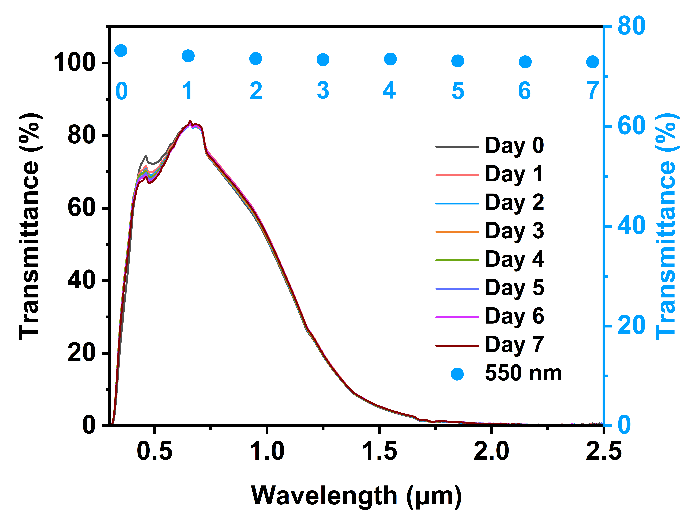
**

**Figure S9.** Solar transmittance variation of the EC glass during one week of exposure under 500 mW/cm² ultraviolet irradiation (measured at one-day intervals), including the transmittance change at the characteristic wavelength of 550 nm.

**
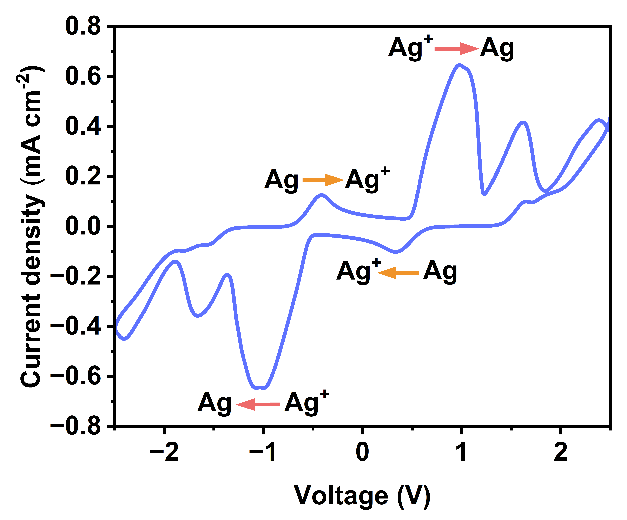
**

**Figure S10.** CV of the EC glass after one week of exposure to 500 mW/cm² UV irradiation, measured at a scan rate of 10 mV/s.

**
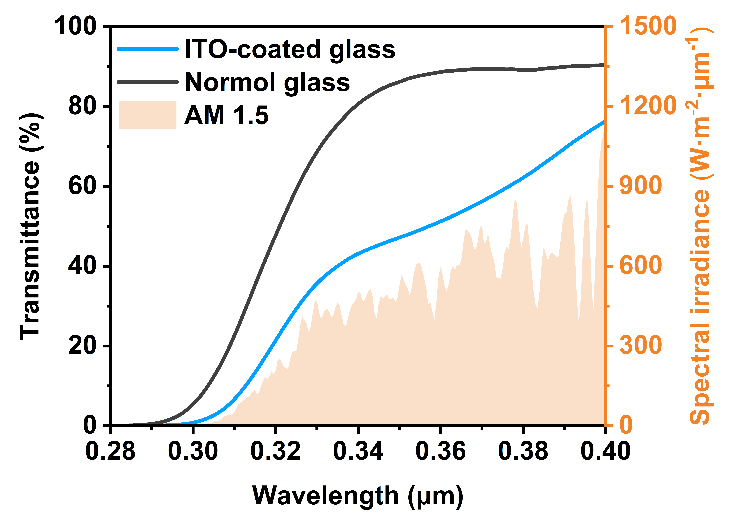
**

**Figure S11.** Difference in transmittance between ITO-coated glass and normal glass in the ultraviolet range.

**Note S4. Calculation Methods for Solar and MIR Spectra**

To quantitatively evaluate the spectral properties of the EC glass, the average solar reflectance and average mid-infrared (MIR) emissivity under different operational modes were calculated by weighting according to the solar radiation intensity (AM 1.5) and blackbody radiation intensity (300 K), respectively ^[3]^.

The average reflectance (*γ*_ave_) is defined as the ratio of the reflected energy to the incident energy in the wavelength range of 0.3-2.5 μm, considering both the solar radiation intensity *I*_AM1.5_(*λ*) and the spectral reflectance *γ*(*λ*).

The total incident solar energy (*E*_in_) is obtained by integrating the solar radiation intensity as a function of wavelength:

$E_{in}=\int_{0.3\text{μm}}^{2.5\mu m} I_{\text{AM}1.5}\left( \lambda\right)d\lambda$ (S1)

The total reflected solar energy (*E*_refl_) is calculated by integrating the product of the spectral reflectance and the solar radiation intensity over the same wavelength range:

$E_{refl}=\int_{0.3\text{μm}}^{2.5\text{μm}} I_{\text{AM}1.5}\left( \lambda\right)\cdot\gamma\left( \lambda\right)d\lambda$ (S2)

The average reflectance (γₐᵥₑ) is then defined as the ratio of total reflected energy to total incident energy:

$\gamma_{ave}=\frac{E_{in}}{E_{refl}}$ (S3)

Similarly, the average emissivity (*ε*_ave_) in the MIR range (2.5-20 μm) is calculated in a similar manner, but considering the spectral distribution of the blackbody radiation intensity *I*_BB_(*T*, *λ*).

The total blackbody radiation intensity (*E*_BB_) is obtained by integrating *I*_BB_(*T*, *λ*) over the wavelength range of 2.5-20 μm:

$E_{BB}=\int_{2.5\text{μm}}^{20\text{μm}} I_{\text{BB}}\left( T\text{, }\lambda\right)d\lambda$ (S4)

The actual radiation intensity (*E*_emit_) of the object is calculated by integrating the product of the spectral emissivity *ε*(*λ*) and the blackbody radiation intensity *I*_BB_(*T*, *λ*) over the wavelength range of 2.5-20 μm:

$E_{emit}=\int_{2.5\text{μm}}^{20\text{μm}} I_{\text{BB}}\left( T\text{, }\lambda\right)\cdot\varepsilon\left( \lambda\right)d\lambda$ (S5)

The average emissivity (*ε*_ave_) is defined as the ratio of the actual emitted radiation to the total blackbody radiation:

$\varepsilon_{ave}=\frac{E_{emit}}{E_{BB}}$ (S6)

The average emissivity of the EC glass within the atmospheric window for radiative cooling (8-13 μm) is calculated following a similar method as that for the average MIR emissivity.

**Note S5. Visible Color Uniformity of the EC Glass**

To evaluate the visible color uniformity of the EC glass in its initial state and after coloration-bleaching cycles, photographs were taken under a uniformly illuminated blue background, as shown in Figures S12a and S13a. To quantitatively assess the visible color uniformity of the images, selected regions of the color photographs (in RGB format) were converted into the Lab color space for analysis. The Lab color space consists of L (lightness), a (red-green component), and b (yellow-blue component), and is widely used to quantify perceived color differences by the human eye ^[4]^.

First, the RGB values were transformed into the CIE XYZ color space using a standard matrix, and subsequently into Lab values, yielding pixel-wise data in the L, a, and b channels. For each channel, the range (difference between maximum and minimum values) was calculated to reflect the variation in lightness (L) and chromaticity (a, b). To evaluate color uniformity, pixel-wise color differences were computed in the Lab space, from which the average color difference (overall uniformity) and the standard deviation of color difference (degree of variation) were derived.

Visible color uniformity analysis of the EC glass in the initial state:

- L channel range (6.85): Minimal variation within the 0-100 scale, indicating a uniform distribution of lightness.
- a channel range (9.14) and b channel range (7.67): Low chromatic variation within the -128 to 127 scale, suggesting consistent hue distribution.
- Average color difference (ΔE = 2.26): Close to the perceptual threshold for the human eye (ΔE ≈ 2-3), indicating high color uniformity with negligible perceived differences.
- Color difference standard deviation (1.65): Low, implying stable and consistent color variation.

Visible color uniformity analysis of the EC Glass after coloration-bleaching cycles:

- L channel range (12.97): Within the 0 to 100 range, the variation in lightness is small (slightly larger than that in the initial state), still indicating a relatively uniform lightness distribution.
- a channel range (8.60) and b channel range (7.44): Low chromatic variation within the -128 to 127 scale, suggesting consistent hue distribution.
- Average color difference (ΔE = 2.27): Close to the perceptual threshold for the human eye (ΔE ≈ 2-3), indicating high color uniformity with negligible perceived differences.
- Color difference standard deviation (1.68): Low, implying stable and consistent color variation.

This analysis indicates that the EC glass exhibits uniform color distribution in the visible spectrum both in its initial state and after cycling, with color differences that are barely perceptible to the human eye.


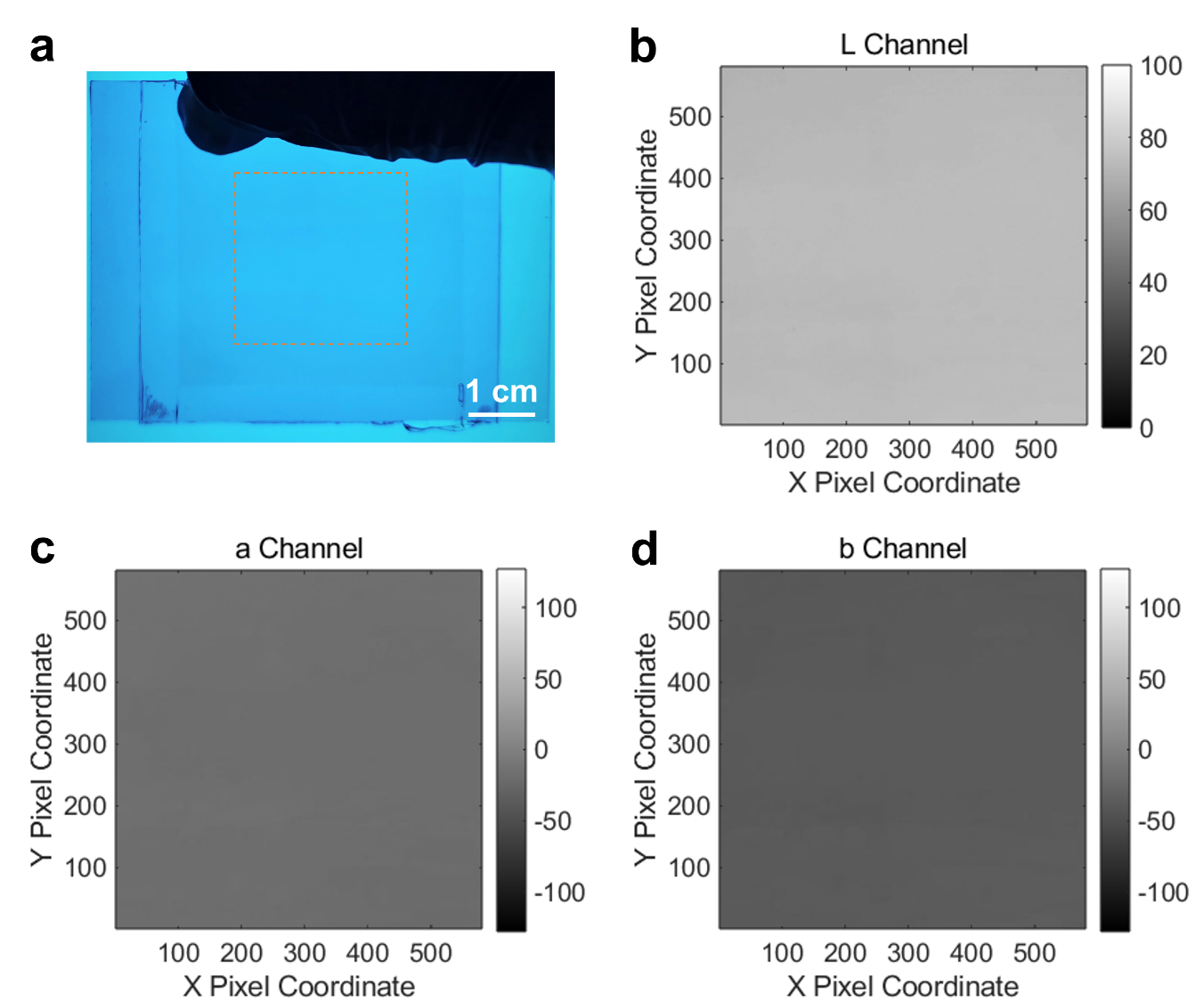


**Figure S12.** (a) Photograph of the EC glass in its initial state under a uniform backlight source and the selected region. (b) Pixel data of the L channel for the selected region. (c) Pixel data of the a channel for the selected region. (d) Pixel data of the b channel for the selected region.

**
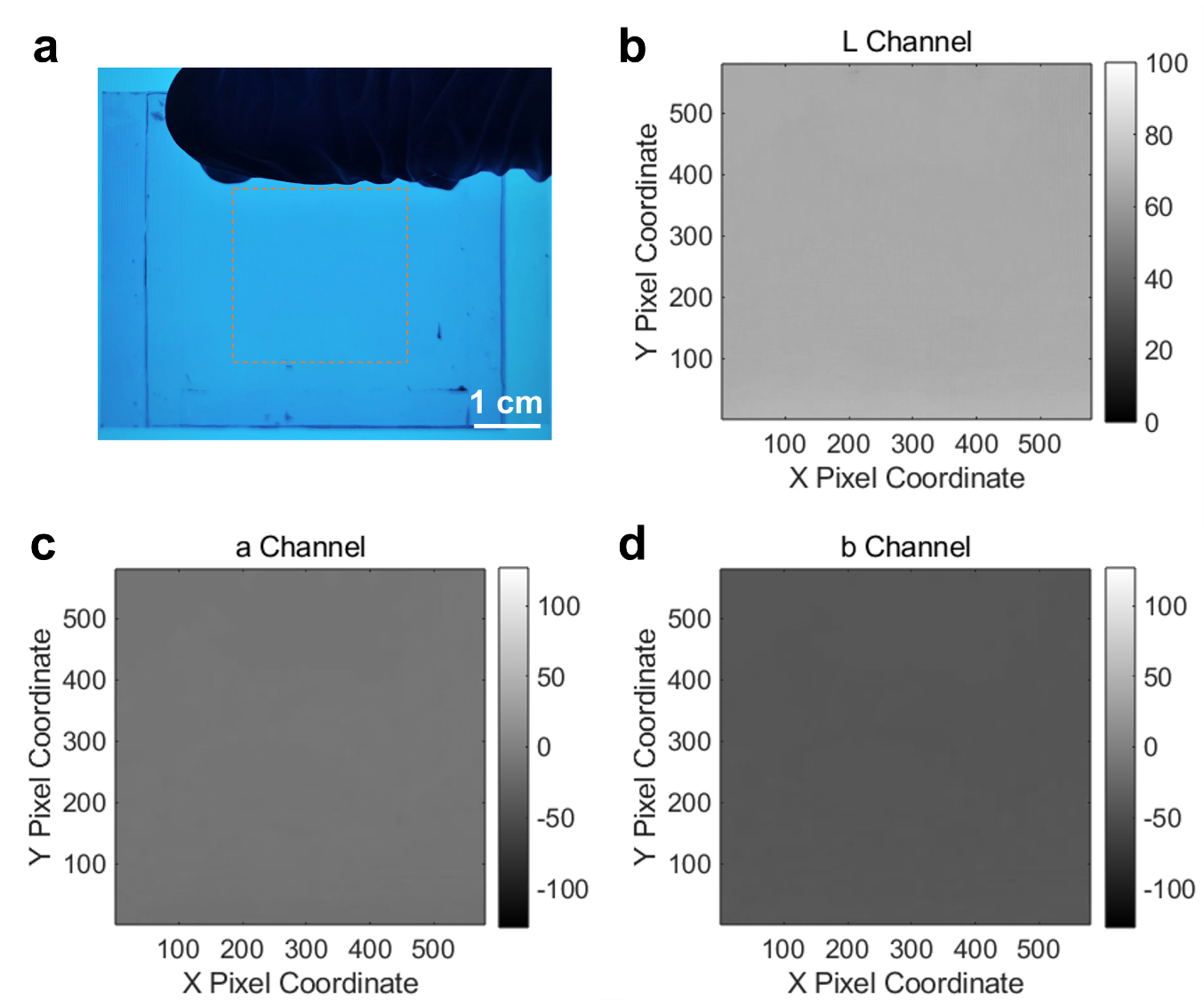
**

**Figure S13.** (a) Photograph of the EC glass after the coloration-bleaching cycles under a uniform backlight source and the selected region. (b) Pixel data of the L channel for the selected region. (c) Pixel data of the a channel for the selected region. (d) Pixel data of the b channel for the selected region.

**
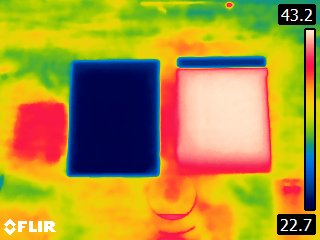
**

**Figure S14.** Thermal imaging of two identical EC glass samples on a 50°C constant temperature heating platform: one with the quartz glass surface facing up and the other with the ITO film facing up.

**Note S6. Intermediate Optical States of the EC Glass**

Given that the activation voltage for Ag reduction shown in the CV curve is greater than 1 V, we applied a series of stepwise voltages (-1.1 V, -1.3 V, -1.5 V, -1.7 V, -1.9 V, -2.1 V, and -2.3 V, each maintained for 600 seconds) and monitored the transmittance change of the EC glass at 633 nm in real time to study its intermediate optical states. As shown in Fig S15, the initial transmittance of the EC glass at 633 nm is 83.3%. Based on the change in transmittance, the threshold voltage for triggering Ag deposition is around -1.3 V. With the application of progressively larger voltages, the transmittance of the EC glass exhibited distinct variations. It is noteworthy that under an applied voltage of -1.7 V, the transmittance of the EC glass remained relatively stable at 63.8%, indicating the presence of a relatively stable intermediate optical state. Depending on user needs and specific application scenarios (e.g., during transitional seasons), this intermediate state could help balance reduced cooling demands with improved daylighting capabilities. When the applied voltage exceeds -2.1 V, the transmittance of the EC glass continues to decrease and becomes difficult to stabilize at an intermediate optical state, eventually reaching a high-reflectance state. The existence of an intermediate optical state expands the potential application scenarios of the EC glass developed in this study and further enhances its prospects for practical applications.


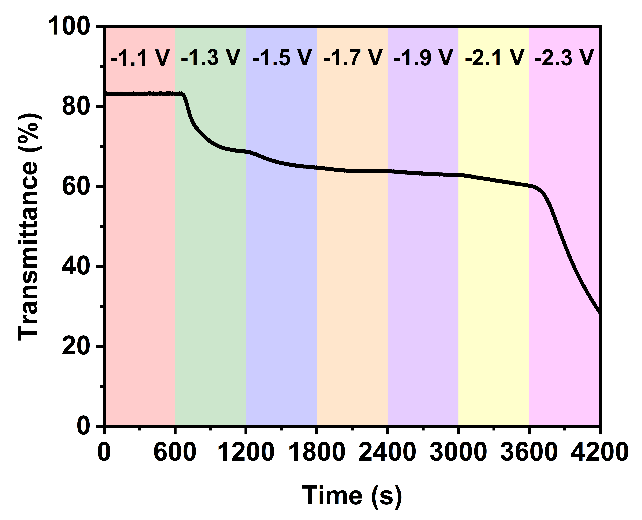


**Figure S15.** Apply stepwise varying voltages to the EC glass and monitor its transmittance at 633 nm in-situ.

**Note S7. Coloration efficiency of the EC glass**

Coloration efficiency (CE) is a key parameter for evaluating electrochromic performance, as a high CE typically indicates advantages such as rapid response and low energy consumption. It can be calculated using the following equation ^[5]^:

$CE=\frac{\Delta OD}{\Delta Q}=\frac{log({T_{b}}/{T_{c})}}{\Delta Q}$ (S7)

where *ΔOD* is the change in optical density, *T_b_* and *T_c_* ​are the transmittances in the bleached and colored states, respectively, and *ΔQ* is the charge density, which can be obtained by integrating the area under the chronoamperometric curve.


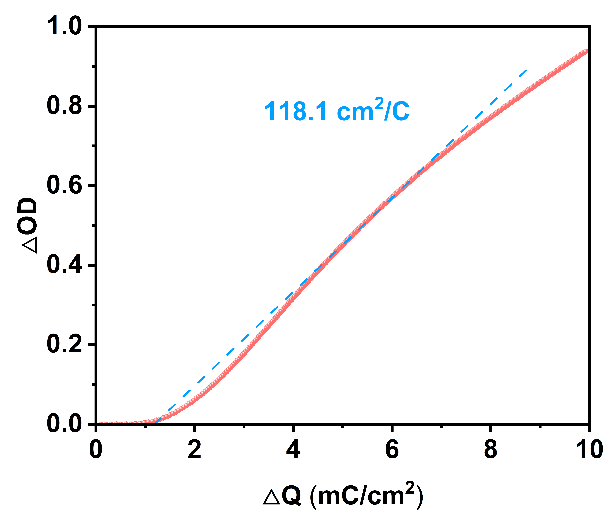


**Figure S16.** Coloration efficiency of the EC glass.

**

**

**Figure S17.** Change in reflectance of the EC glass at 633 nm over time under continuous application of a -2.5V pulse voltage.


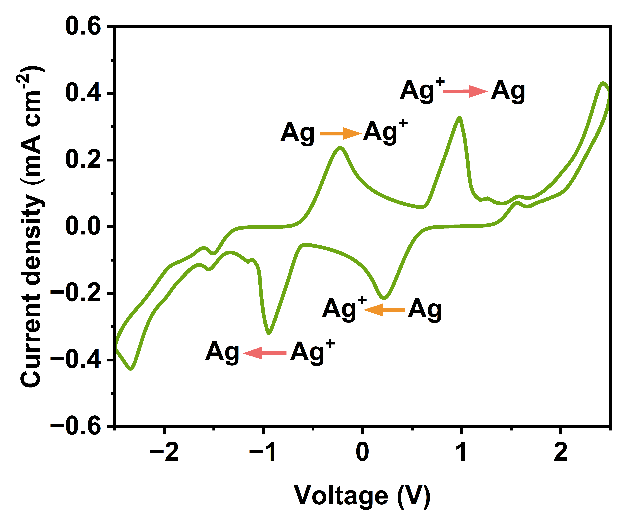


**Figure S18.** CV of the EC glass tested using a two-electrode system after 1000 coloration-bleaching cycles, measured at a scan rate of 10 mV/s.

**
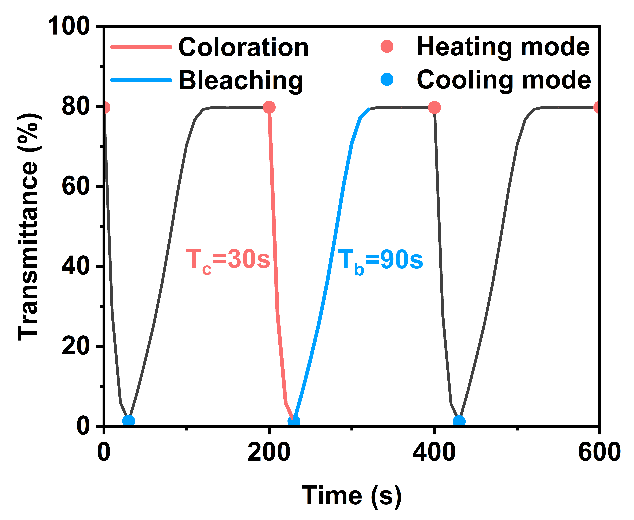
**

**Figure S19.** Optical response of the EC glass to voltage during the first 600 seconds of the coloration-bleaching cycles.

**
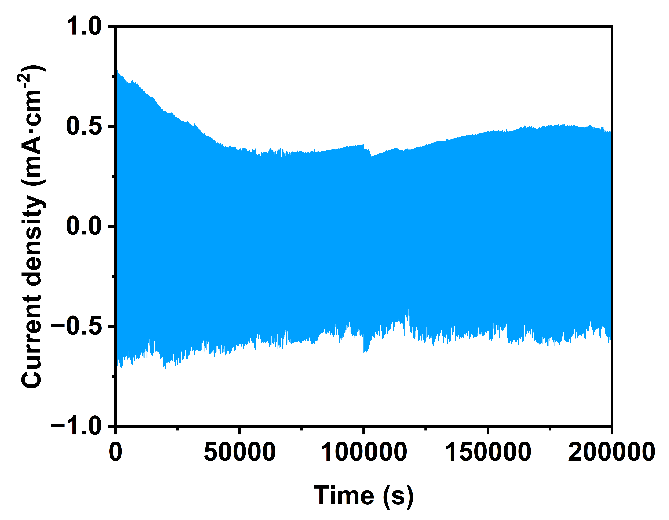
**

**Figure S20.** Current-time relationship corresponding to the entire coloration-bleaching cycles.

**
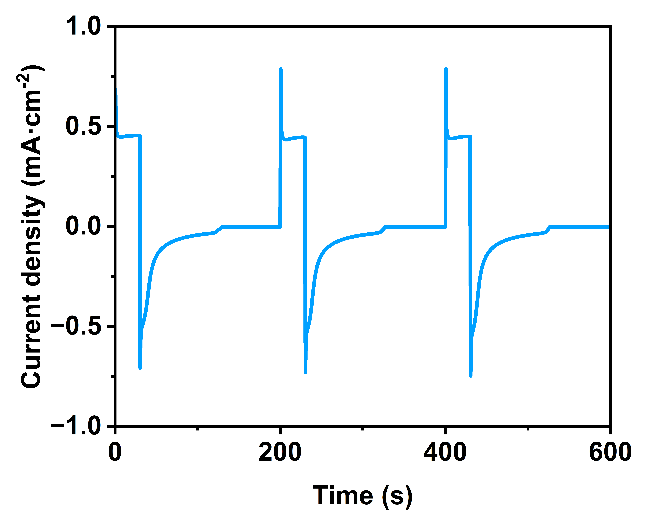
**

**Figure S21.** Current-time relationship corresponding to the first 600 seconds of the coloration-bleaching cycles.

**
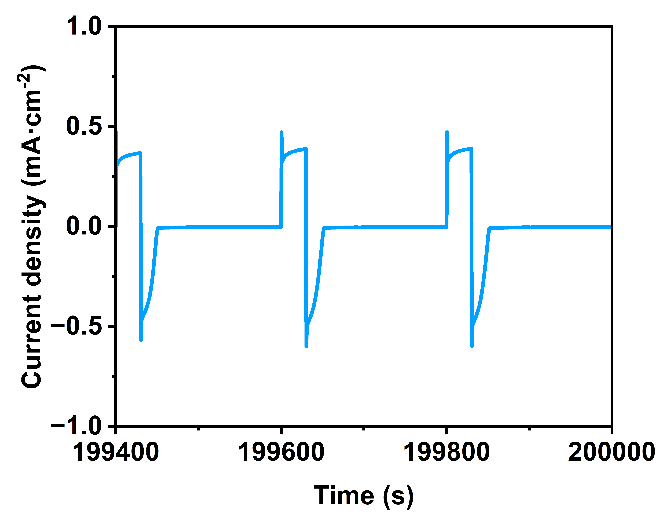
**

**Figure S22.** Current-time relationship corresponding to the last 600 seconds of the coloration-bleaching cycles.


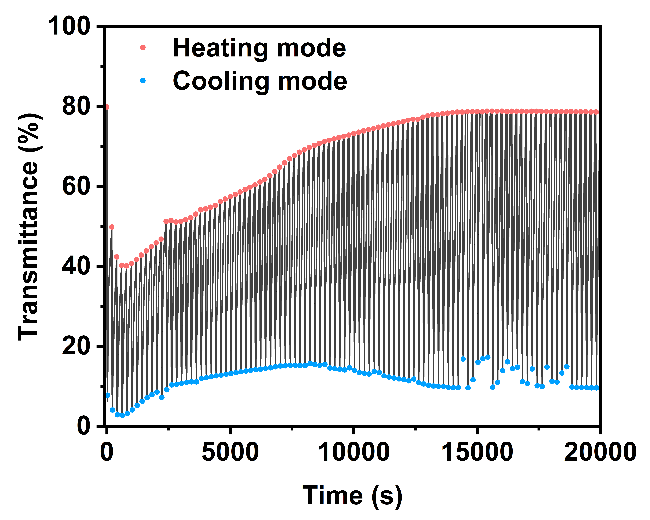


**Figure S23**. The coloration-bleaching cycling stability of the EC glass without plasma cleaning and MPTMS modification was evaluated at 633 nm over a duration of 20,000 seconds under a voltage sequence of -2.5 V for 30 s followed by 0 V for 170 s.

**

**

**Figure S24.** Solar absorption rate of the black solar absorbing layer on the back of the EC glass.

**
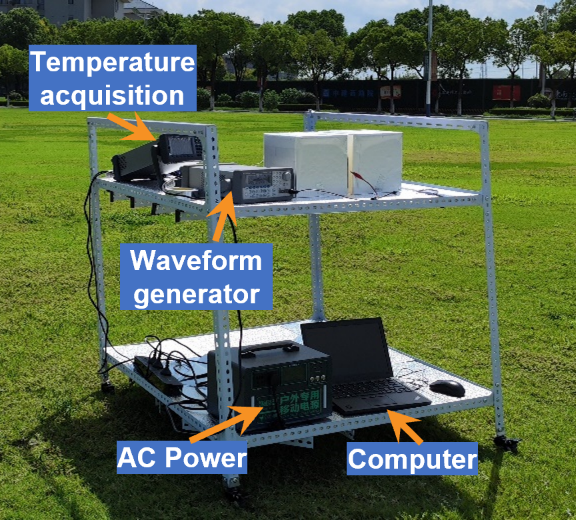
**

**Figure S25.** Photograph of the outdoor experimental setup.

**
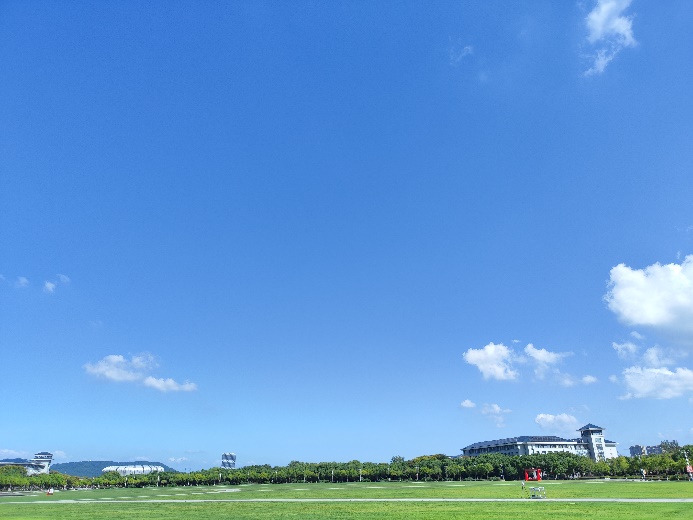
**

**Figure S26.** Sky conditions on the day of the outdoor experiment.

**Note S8. Automated Control of the EC Glass**

A simple layered system architecture can be designed to manage the operational state of the EC glass based on ambient temperature, comprising sensors, a controller, and actuators, as illustrated in Figure S27. The temperature sensor must exhibit sufficient accuracy, as the mode-switching behavior of the EC glass is temperature-dependent. The main controller is responsible for processing sensor data, determining the appropriate operating mode, and coordinating the voltage application and motor rotation. The actuators include the EC driving circuit and the stepper motor driver module.

The control logic operates as follows: when the temperature detected by the sensor exceeds 20 °C, the controller receives the feedback and activates the EC driving circuit to apply a sustained voltage of -2.5 V, initiating Ag deposition. Simultaneously, the controller drives the stepper motor to perform a forward planar rotation of 180° within 30 seconds, thereby orienting the glass side of the EC device outward and switching to the radiative cooling mode. It is important to ensure precise 180° rotation of the stepper motor, which may require position feedback for verification. This can be implemented using encoders or limit switches that send signals back to the controller. Similarly, when the temperature falls below 20 °C, the controller removes the applied voltage to dissolve the Ag reflective layer and commands the stepper motor to rotate backward by 180° within 30 seconds, reorienting the ITO-coated side of the EC glass to face outward and switching to the solar heating mode. Again, accurate rotation with position feedback is necessary. Furthermore, considering the close correlation between the EC glass operating state and solar irradiation, a power management system integrating solar panels with lithium batteries can be employed to further reduce energy consumption.


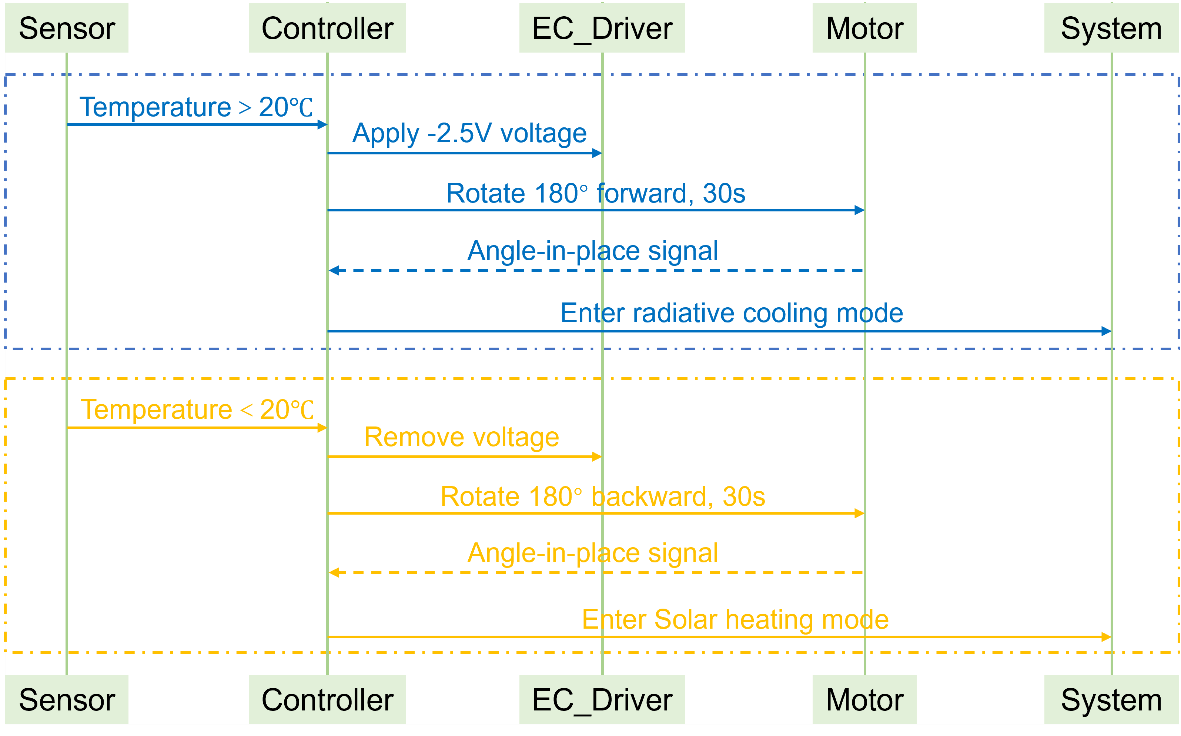


**Figure S27.** Hierarchical Architecture and Control Logic of the EC Glass.


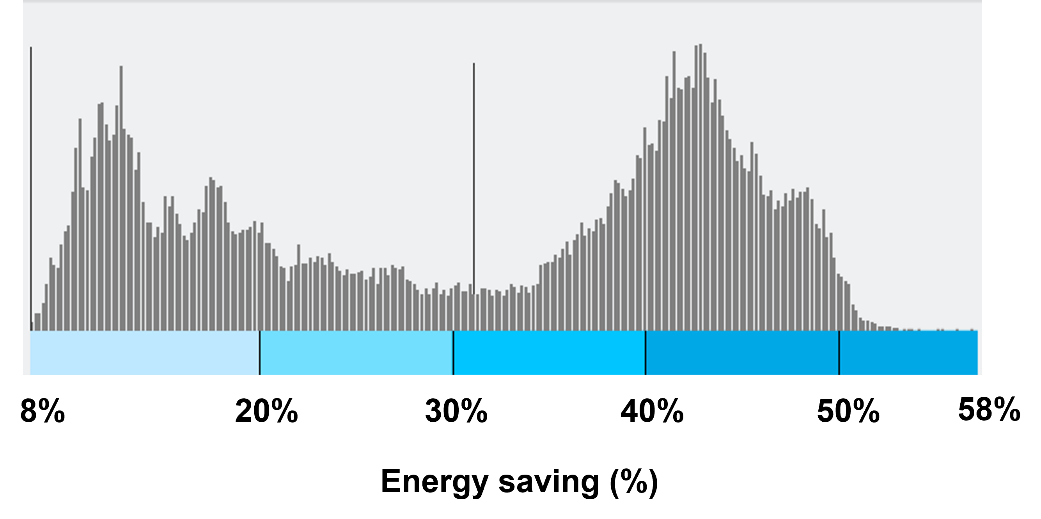


**Figure S28.** Distribution of annual heating, ventilation, and air conditioning (HVAC) energy savings percentage from the global application of the EC glass.


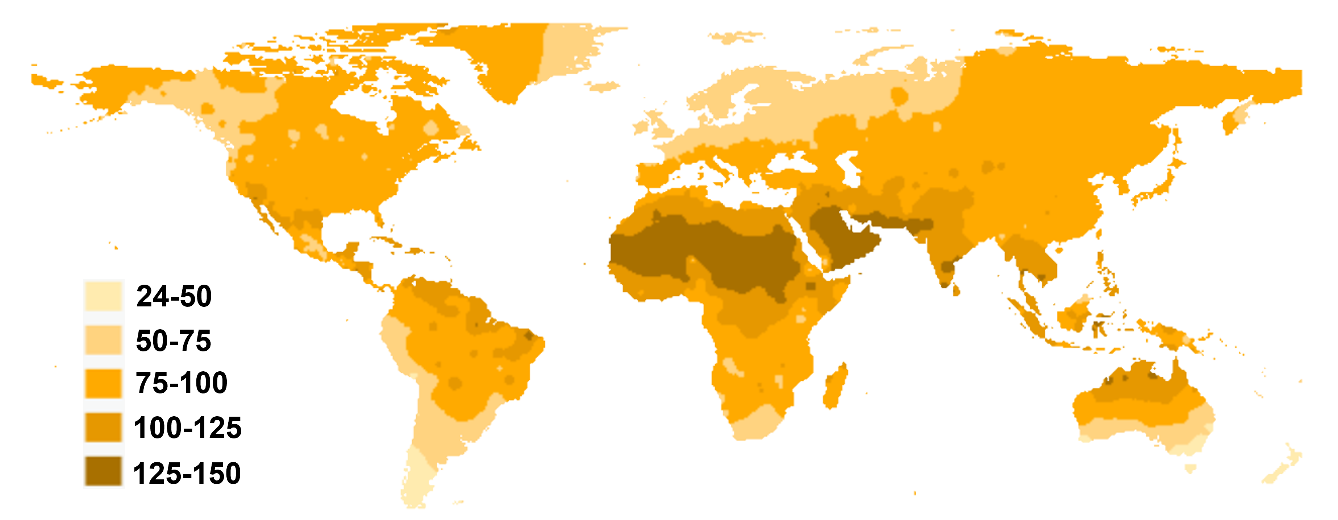


**Figure S29.** Annual HVAC energy savings from the global application of the EC glass, considering local climate conditions.


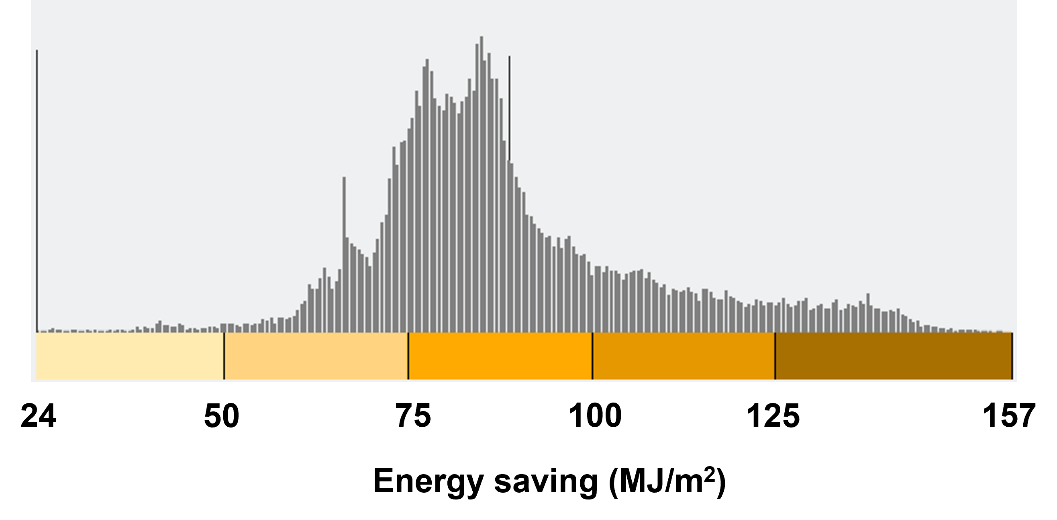


**Figure S30.** Distribution of annual HVAC energy savings from the global application of the EC glass.

**Table S1.** Parameters of the medium office building model used in the EnergyPlus simulation.

| **Parameters** | **Value** |
| --- | --- |
| Total building area | 4982.19 m^2^ |
| Roof area | 1660.73 m^2^ |
| Roof insulation | 4.31 (m^2^·K)/W |
| Number of floors | 3 |
| Window SHGC | 0.25 |
| Window U-factor | 2.41 W/(m^2^·K) |
| Window-to-wall ratio | 33% |

**Table S2.** Parameters of the normal glass skylight used in the model building.

| **Parameters** | **Value** |
| --- | --- |
| Roughness | Smooth |
| Thickness | 0.0072 m |
| Thermal conductivity | 0.9 W/(m·K) |
| Solar transmittance | 70% |
| Solar reflectance | 15% |
| Infrared absorptance | 84% |

**Table S3.** Parameters of the dynamic EC glass skylight used in the model building.

| **State** | **Solar Transmittance** | **Solar Reflectance** | **Infrared Absorptance** |
| --- | --- | --- | --- |
| Heating mode | 61.2% | 19.9% | 10.8% |
| Cooling mode | 0.09% | 87.9% | 90.6% |

**Table S4.** Annual HVAC Energy Savings Analysis of 15 Typical Cities.

| City | HVAC Energy Savings (MJ/m2) | HVAC Energy Savings Percentage (%) |
| --- | --- | --- |
| Buenos Aires | 53.13 | 38.72 |
| Sydney | 46.35 | 39.45 |
| São Paulo | 58.48 | 40.98 |
| Vancouver | 66.61 | 34.09 |
| London | 78.81 | 26.42 |
| Beijing | 85.27 | 35.30 |
| Shanghai | 87.82 | 44.26 |
| Cairo | 101.01 | 49.62 |
| Paris | 78.45 | 38.51 |
| Mumbai | 92.98 | 35.15 |
| Tokyo | 82.10 | 43.10 |
| Singapore | 86.61 | 32.73 |
| Los Angeles | 64.71 | 52.47 |
| New York | 81.62 | 35.29 |
| Cape Town | 49.49 | 43.36 |

**References**

1. S. Araki, K. Nakamura, K. Kobayashi, A. Tsuboi, N. Kobayashi, Adv. Mater. 2012, 23, OP122.
2. C. Park, S. Seo, H. Shin, B. D. Sarwade, J. Na, E. Kim, Chem. Sci. 2015, 6, 596.
3. F. Fan, Q. Xu, H. Pan, H. Tang, C. Guo, Z. Dai, D. Zhao, Int. J. Heat Mass Transfer 2025, 242, 126872.
4. T. Huang, Q. Chen, J. Huang, Y. Lu, H. Xu, M. Zhao, Y. Xu, W. Song, ACS Appl. Mater. Interfaces 2023, 15, 16277.
5. H. Zhang, F. Sun, J. Feng, H. Ling, D. Zhou, G. Gao, S. Wang, F. Su, Y. Tian, Y. Tian, Cell Rep. Phys. Sci. 2022, 3.
